# Fabrication of 3-Dimensional-Printed Bilayered Scaffold Carboxymethyl Chitosan/Oxidized Xanthan Gum, Biphasic Calcium Phosphate for Osteochondral Regeneration

**DOI:** 10.34133/bmr.0186

**Published:** 2025-04-09

**Authors:** My N.-H. Nguyen, Binh T. Vu, Dung M. Truong, Thanh D. Le, Thanh-Tuyen T. Vo, Toi V. Vo, Thi-Hiep Nguyen

**Affiliations:** ^1^ Tissue Engineering and Regenerative Medicine Department, School of Biomedical Engineering, International University, Ho Chi Minh City, Vietnam.; ^2^ Vietnam National University, Ho Chi Minh City, Vietnam.; ^3^ Biotechnology Center of Ho Chi Minh City, Ho Chi Minh City, Vietnam.; ^4^ Thong Nhat Hospital, Ho Chi Minh City, Vietnam.

## Abstract

Cartilage tissue regeneration remains challenging due to the tissue’s poor self-healing capacity, attributed to its hypocellular and avascular nature, which limits nutrient delivery to the defect site and complicates healing. Traditional methods often utilize the subchondral tissue layer to improve nutrient exchange through its vascular network, although these approaches have limitations. To address these issues, 3-dimensional (3D) printing has been employed to create the bilayered scaffold that mimics the complex structure of osteochondral tissue. In this study, the *N*,*O*-carboxymethyl chitosan (NOCC) and oxidized xanthan gum (OXG) hydrogel was fabricated for the cartilage layer due to its similarity to the native cartilage structure, while the biphasic calcium phosphate (BCP) incorporation enhanced the osteoconductivity to promote new bone growth for osteochondral tissue regeneration. Various characterization tests, including compression strength, scanning electron microscopy analysis, and biological properties, were conducted to evaluate and balanced to achieve the highest regenerative capacity for implantation. No cytotoxicity was caused, while the in vitro testing highlighted that the addition of BCP considerably supported cellular behavior on the scaffold and improved the regeneration rate. With 60% BCP content, the 3D scaffold demonstrated a high osteochondral tissue regeneration rate, as evidenced by visual inspection, x-ray imaging, and histological analysis, outperforming other experimental models.

## Introduction

Articular cartilage injury has emerged as a prevalent global health issue in recent years, posing a substantial treatment challenge in orthopedics and sports medicine [1]. Unlike other types of tissue, it is difficult to have the whole cartilage defect well regenerated. Hypocellularity and avascularity, along with the lack of neural and lymphatic networks, impede the natural healing process, contributing to chronic wound development and potentially exacerbating conditions like osteoarthritis and rheumatoid arthritis [2]. While several traditional clinical techniques have been documented, their persistent limitations, including fibrocartilage formation, limited donor tissue, and low mechanical strength, drive the urgent demand for more effective cartilage tissue regeneration therapies [3].

According to Chen et al. [4], cartilage tissue regeneration should be approached by targeting the underlying subchondral bone layer, where the nutrient supply and cellular sources from the vascular network facilitate the simultaneous remodeling of both subchondral bone and cartilage, especially in the more complex structure of osteochondral defect case. Considering that injectable hydrogels offer a promising method by the easier handling, potential for in situ application, and compatibility with irregularly shaped defects, they still possess some limitations in terms of mechanical strength, nutrient exchange, or in vivo stability that may not be appropriate for osteochondral tissue regeneration application [5]. Owing to the hydrogel-based structure with high water fraction, the injectable hydrogel illustrated the low mechanical force without additional reinforcement strategies and fast degradation rate, which makes the scaffold inappropriate for osteochondral tissue regeneration. According to Zhou et al. [6], the injectable hydrogel may create a nano-sized or submicrometer porous structure within a dense polymer network that holds the inappropriateness for nutrient and oxygen transport within the migration and ingrowth of cells. Besides the superior advantage of irregular shape filling in, the major challenge is the shortage of macroporosity formation—the key role in the vascularization process, since the hydrogel liquid is filled over completely on the wound site and then the sol–gel process takes place within an undefined shape [7]. Recently, the injectable hydrogel has been utilized to incorporate with other biomaterials for the 3-dimensional (3D) printing technique development as the powerful way to replicate the 3D bilayered structure of native cartilage and subchondral bone’s extracellular matrices (ECMs) within distinguished mechanical and biological properties [8]. By providing a wide range of macroporosity along with microporosity corresponding to each tissue layer’s characteristics, it optimizes the mechanical and biological properties of the scaffold, as well as induces the vascularization process to enhance the efficacy of bone tissue regeneration. With the ability to assemble diverse biomaterials from heterogeneous inorganic/organic compounds, 3D printing technology provides a fast, high-precision, and robust strategy to fabricate structurally diversified scaffolds to individual patients regarding parameters such as shape, dimensions, and internal structural organization from a single precursor slurry by a one-step process through computer [9]. Among common techniques of 3D printing, extrusion-based 3D printing stands out as the most versatile, rapid, scalable, and cost-efficient method [10].

In recent years, hydrogels have been widely used as the bioink in cartilage defect repair and regeneration since their 3D hydrophilic polymer networks are similar to the articular cartilage ECM. Owing to the native structure of glycosaminoglycans (GAGs) and hyaluronic acid (HA), chitosan (CS) acts as a suitable candidate chosen as a component of the bioink for cartilage regeneration [11]. By chemical modification to *N*,*O*-carboxymethyl chitosan (NOCC), its amine group is able to crosslink with the aldehyde group of modified xanthan gum (XG) [oxidized XG (OXG)] to form Schiff base crosslinking, creating a microenvironment that favors the stabilization of proteins and allows formation of hydrogels. It is also reported that Schiff base reaction creates less toxic chemicals and byproducts, resulting in successive improvement in physicochemical properties such as solubility, swelling, and extended successful intrinsic self-healing ability at room temperature while maintaining a good mechanical strength to support the cartilage tissue regeneration [12]. In addition to facilitate the osteogenic formation for the bone tissue regeneration process, bioactive factor supplementation into the system are crucial. Biphasic calcium phosphate (BCP), according to Bouler et al. [13], is considered as a gold standard bio-ceramic in bone reconstructive surgery due to the stable phase of hydroxyapatite (HAp) and the soluble phase of β-tricalcium phosphate (β-TCP), therefore demonstrating the controllable degradation as the native bone tissue and excellent osteoconductive property toward new bone ingrowth. With the incorporation of BCP into the NOCC-OXG hydrogel system, CS overcomes the brittleness of BCP by acting as the binder, and subsequently, BCP could reinforce and stiffen the CS matrix by the hydrogen bonding formation within the strong coordination bond between the NH_2_ of NOCC and Ca^2+^ of BCP [14]. Additionally, a uniform layer of calcium phosphate precipitation is produced, which served as the crucial factor in inducing the osteoconductive property for better subchondral bone layer regeneration [15].

Up to now, cartilage tissue regeneration remains a considerable challenge for scientists as a prevalent global health concern due to its popularity and low self-healing capacity, thus highlighting the urgent need for effective appropriate treatment. Extensive research has been conducted to address these issues, with 3D printing technology standing out as a more effective approach for fabricating bilayered scaffolds that closely mimic the osteochondral tissue ECM, thereby enhancing regeneration efficacy. Scaffold fabrication from hydrogels or multi-layered has been widely investigated. Nevertheless, there has been no reported combination between NOCC and OXG incorporated with BCP for the 3D bilayered scaffold printing toward the cartilage–bone tissue engineering application. This study aims to fabricate a 3D bilayered scaffold, composed of NOCC-OXG for the cartilage layer and BCP-incorporated material for the subchondral bone layer, to mimic the cartilage–bone tissue structure and leverage osteoconductive properties for bone regeneration. NOCC-OXG and NOCC-OXG-BCP hydrogels are fabricated and then optimized to investigate the most suitable ratio among components based on native tissue’s physicochemical properties as well as in vitro testing for the biocompatibility and bioactivity. Then, in vivo study on rabbit model is evaluated to investigate the regeneration capacity when compared to osteochondral autologous transplantation (OAT) and healthy rabbit defect model.

## Materials and Methods

### Experimental design

**Fig. 1.**
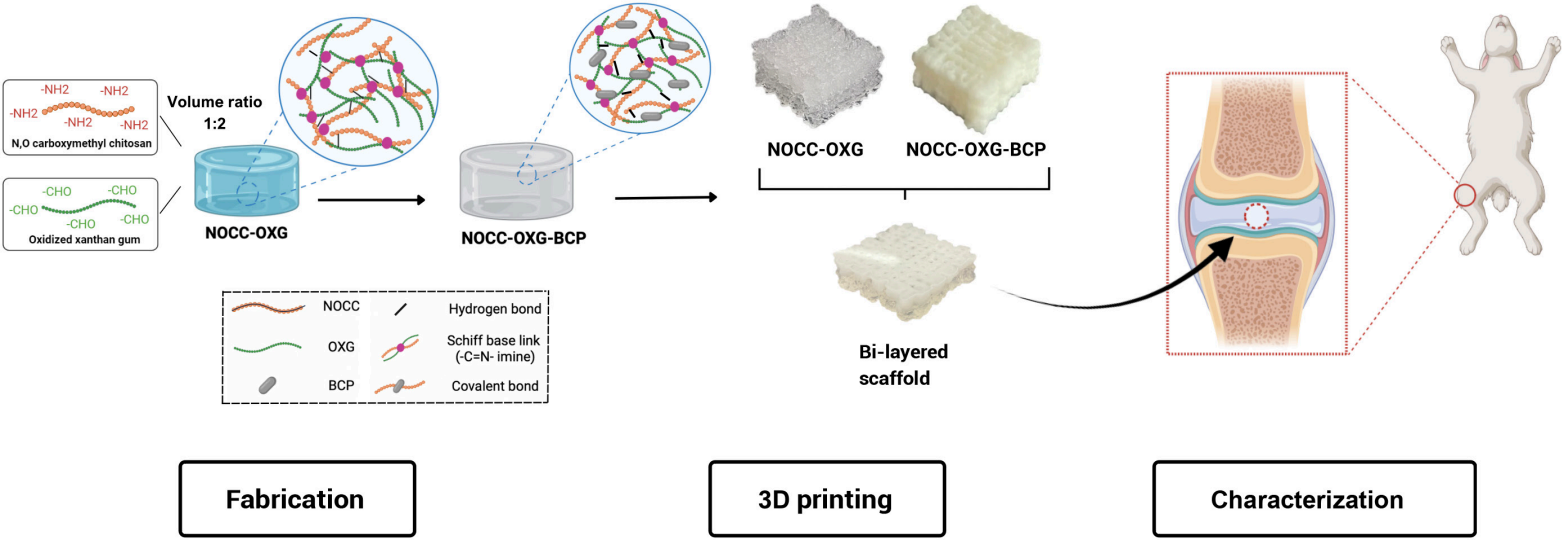
Given the challenges and pressing need in osteochondral tissue regeneration, a bilayered scaffold was chosen to ensure that each layer’s properties are appropriately matched.

The aim of this study is to develop a hydrogel-based bilayered scaffold using 3D printing for cartilage tissue regeneration (Fig. 1). Specifically, NOCC and OXG will be employed for the regeneration of cartilage tissue, and BCP will be incorporated to enhance osteoconductivity and support osteochondral tissue regeneration, which is critical for the seamless integration with the cartilage layer. Various ratios among components have been fabricated and subjected to a series of characterization tests, including scanning electron microscopy (SEM), energy-dispersive x-ray spectroscopy (EDS), Fourier transform infrared (FTIR) spectroscopy, x-ray diffraction, gelation time, pore size and porosity measurements, swelling ratio, and degradation rate analysis in order to give the most optimal formula for further application. Through cytotoxicity, live/dead assays, and proliferation rate evaluation, the biocompatibility of the scaffold was revealed and demonstrated the role of the hydrogel-based scaffold’s support on both 2D and 3D culturing. Through in vitro and in vivo experiments, corresponding scaffolds were illustrated to facilitate the osteogenic and chondrogenic differentiation, supportive for the cartilage tissue healing process by the gross visualization, x-ray imaging, and histological analysis with the purpose of assessment of experimental groups’ regenerative efficiency when compared to other groups.

### Materials

CS (from shrimp shells; degree of deacetylation ≥ 75%) was purchased from Sigma-Aldrich (USA). XG and chloroacetic acid were purchased from HiMedia Laboratories Pvt. Ltd. (India). Calcium nitrate tetrahydrate [Ca(NO3)_2_.4H_2_O], diammonium hydrogen phosphate [(NH_4_)_2_HPO_4_], and isopropyl alcohol were purchased from Guanghua Sci-tech, China. Sodium metaperiodate (NaIO_4_, ≥99%) was purchased from Thermo Fisher Scientific Inc. (UK). Sodium hydroxide (NaOH), hydrochloric acid (HCl), and ethylene glycol were purchased from Xilong Chemical Ltd. (China). Dialysis tubing cellulose membrane [D9652, molecular weight cutoff (MWCO) of 12 to 14 kDa] and phosphate-buffered saline (PBS) tablets were purchased from Sigma-Aldrich Co. LLC (USA). Trypsin/ethylenediaminetetraacetic acid (EDTA), Dulbecco’s modified Eagle’s medium (DMEM), fetal bovine serum (FBS), and antibiotics (penicillin and streptomycin) (100 ml, 100×) were purchased from Thermo Fisher Scientific Inc. (USA).

### Fabrication of NOCC-OXG/NOCC-OXG-BCP 3D-printed scaffold

#### Synthesis of NOCC, OXG, and BCP

NOCC was synthesized with some adjustments as have been mentioned by previous research [16,17]. In summary, 2 g of CS was submerged in 10 ml of isopropyl alcohol at room temperature. Subsequently, 10 ml of 13 N NaOH was added, followed by allowing the mixture to stand for 1 hour. Then, 5.2 g of chloroacetic acid was dissolved in 10 ml of isopropyl alcohol, and this solution was incrementally added to the previous mixture in 5 equal portions at 5-min intervals, with the reaction being heated at 60 °C. The resulting mixture was filtered to collect the solid residue product. Due to the high pH of the solid sample, it was fully dissolved in distilled water and neutralized using HCl. The neutral solution was then dialyzed for 3 d using a dialysis bag (MWCO, 12 to 14 kDa), with the water being replaced 3 times daily. Following dialysis, the solution was frozen overnight and subsequently lyophilized to obtain the final product. Finally, the NOCC was stored in a vacuum oven at room temperature to prevent oxidation.

OXG was fabricated according to previous studies with slight modifications [18]. Briefly, 200 ml of 0.6% (w/v) XG solution was prepared. Next, 0.28, 0.55, and 0.83 g of sodium periodate (NaIO_4_) were dissolved in 4 ml of distilled water in the dark before being added dropwise into XG solution to prepare OXG with the oxidation levels 1, 2, and 3, respectively. The container was light-protected with aluminum foil and stirred continuously for 3 h. Then, 1 ml of ethylene glycol was added and stirring was kept for 1 hour. Next, the dialysis process was performed using dialysis bag (MWCO, 12 to 14 kDa) against distilled water being replaced 3 times per day. After 9 times, the dialyzed solution was pH adjusted to 7.4 by NaOH. The solution was then kept at −80 °C overnight before being lyophilized to get the final product.

BCP was synthesized by using a water-based precipitation technique, which was carried out according to Bakan [19]. Briefly, 50 g of Ca(NO3)_2_.4H_2_O was dissolved under 60 °C, and 18.34 g of (NH_4_)_2_HPO_4_ aqueous solutions was used as the Ca and P precursors, respectively. After completely dissolving, (NH_4_)_2_HPO_4_ was poured into the Ca(NO3)_2_.4H_2_O solution, which turned into a milky color. Then, pH by 4 M NaOH was adjusted until it is around 7 and maintained for 120 min. After that, the BCP solution was washed 6 times, 3 h apart, and the white precipitates were dried in an oven at 100 °C for 24 h before being ground into fine powders with a mortar and pestle. Finally, the white powders were calcined in a furnace at 800 °C for 6 h.

#### Optimization of NOCC-OXG hydrogel

The NOCC-OXG hydrogel is fabricated first with the purpose of choosing the optimal oxidation level of OXG solution as well as the volume ratios between NOCC and OXG solution. The dry products of NOCC, OXG1, OXG2, and OXG3 were first dissolved in distilled water at the concentration of 20 mg/ml to prepare NOCC-OXG hydrogels. Then, the crosslinked hydrogels were fabricated by mixing NOCC solution with OXG1, OXG2, or OXG3 solution at volume ratios of 1:1, 1:2, 1:3, and 1:4 (Table 1). NOCC and OXG were thoroughly mixed. Briefly, the 2 solutions were mounted into two 1-ml syringes and then blended by being pushed through chambers containing helical static mixers to produce a complete mixture of crosslinked gel, with a final volume of each hydrogel being 600 μl. Through the SEM and compression test, NOCC and OXG2 within the volume ratio 1:2 will be fixed for the NOCC-OXG-BCP fabrication.

**Table 1.** Sample coding of NOCC-OXG scaffold at different oxidation levels of OXG and volume ratios between NOCC-OXG

Volume ratio	NOCC-OXG1	NOCC-OXG2	NOCC-OXG3
1:1	NO1-1	NO2-1	NO3-1
1:2	NO1-2	NO2-2	NO3-2
1:3	NO1-3	NO2-3	NO3-3
1:4	NO1-4	NO2-4	NO3-4

#### Fabrication of NOCC-OXG-BCP hydrogel

For the NOCC-OXG-BCP hydrogel fabrication, the dry products of NOCC and OXG2 were first dissolved in distilled water at the concentration of 20 mg/ml. The BCP powder was prepared at the weight ratio 50%, 60%, and 70% (w/w) of the total hydrogel weight. The BCP powder was first completely dispersed in NOCC solution, and the mixture was then mixed with OXG solution at the ratio of 1:2 to form the NOCC-OXG-BCP hydrogel. Three hydrogel samples were fabricated in total, namely, NO2B5, NO2B6, and NO2B7, and NO2 without the BCP content is regarded as the BCP-free scaffold group, as shown in Table 2.

**Table 2.** Sample coding of NOCC-OXG-BCP scaffold at different contents of BCP powder

	NOCC-OXG	NOCC-OXG-BCP		
BCP content	0% BCP	50% BCP	60% BCP	70% BCP
Sample coding	NO2	NO2B5	NO2B6	NO2B7

#### Fabrication of NOCC-OXG/NOCC-OXG-BCP bilayered 3D-printed scaffold

The fabrication of NOCC-OXG/NOCC-OXG-BCP bilayered 3D-printed scaffolds was meticulously designed using Simplify3D, a sophisticated printing software from 3DPL, and produced with precision by the 3DPL Bioprinter N2 Plus. This process exemplifies the integration of advanced software and printing technology to create complex and functional biomaterials. Briefly, NO2 is representative of the cartilage-regenerating bioink and combined with 3 contents of BCP, which are 50%, 60%, and 70% for the bone-regenerating bioink. Each biomaterial solution was prepared as have been mentioned in the biomaterial’s synthesis section. NOCC solution and BCP content were mixed for complete dissolution first, and then OXG solution was added to the existing system. Hydrogels were kept at room temperature for 30 min and then loaded into a syringe and prepared for the printing process. The operation of the 3D bioprinter was optimized depending on the texture of the gel to reach the balance among the parameters to give out the complete scaffold without damage or interruption. A G22 syringe head with a diameter of 400 μm is used. In detail, 1 × 1 × 1 cm^3^ cube was created (pressure from 1.8 to 3 MPa, printing speed of 650 mm/min, layer height of 0.3 mm, infill percentage of 35%, internal fill pattern of rectilinear, internal angle offsets of 0 and 90, and printing temperature at room temperature). The NOCC-OXG hydrogel is used for the cartilage tissue layer, while the NOCC-OXG-BCP composite is used for the subchondral bone tissue layer.

After the first layer was printed, we continued to print the upper layer to form a bilayered 3D scaffold. Based on the native structure of osteochondral tissue, the subchondral bone layer is beneath the above cartilage layer. Therefore, the NOCC-OXG-BCP layer was printed first as has been mentioned above, then the syringe containing the NOCC-OXG hydrogel was replaced, and the printing process proceeded to print on the existing scaffold with the same setting parameters. Particularly, to match the osteochondral defect of 3 mm in diameter and 3 mm in depth of the in vivo rabbit model, the subchondral bone and cartilage layers are 2 mm and 1 mm in thickness, respectively.

### Characterization of NOCC-OXG-BCP 3D-printed scaffold

#### Gelation time

NOCC, OXG solution [2% (w/v)], and BCP powder are prepared. NOCC and BCP powder are dissolved first and then brought to measure the gelation time. The experiment is performed by using the Modular Advanced Rheometer System, Haake Mars. After running the machine, NOCC-BCP solution and OXG solution are respectively added to the measuring point. *G*′ and *G*″ are measured and then recorded. The experiment was performed in triplicate.

#### FTIR spectroscopy analysis

The FTIR technique was utilized to detect the presence of various functional groups, including phosphate groups in BCP, aldehyde groups in OXG, carboxymethyl groups in NOCC, and Schiff base crosslinks between OXG and NOCC. The analysis was performed using a Vertex 70v FTIR spectrometer (Bruker, USA). Measurements were carried out on freeze-dried samples within the wavenumber range of 4,000 to 600 cm^−1^.

#### X-ray diffraction analysis

The crystalline structure of materials was examined using an x-ray diffractometer (XRD), specifically the XRD D2 PHASER (Bruker, UK). This machine employs Cu Kα radiation with a wavelength (λ) of 1.5406 Å and operates within the 2θ range of 10° to 80° at 25 °C. Phase analysis was conducted on a range of freeze-dried hydrogel samples and solid-phase BCP to identify distinct crystalline phases.

#### Characterization of NOCC-OXG-BCP scaffold surface morphology

SEM was used to observe the cross-sectional surface morphology of the NOCC-OXG hydrogels. Briefly, the crosslinked hydrogels were formed by combining NOCC solution (20 mg/ml) with OXG1, OXG2, or OXG3 solution (20 mg/ml) at 4 ratios (1:1, 1:2, 1:3, and 1:4). Hydrogel samples were prepared and freeze-dried for at least 6 h to yield the scaffolds. Each sample was then sliced to show the interior cross-sectional surface, which was next coated with a thin gold layer by Smart Coater (JEOL, Japan) and observed with a JSMIT100 microscope (JEOL, Japan) at 5 to 10 kV. ImageJ software was employed to analyze the pore size of the hydrogels at 100 discrete points within each sample.

For characterization of the surface morphology of the NOCC-OXG-BCP hydrogel, NOCC-OXG2 with a volume ratio of 1:2 was fixed and combined with BCP at the weight ratios of 20%, 40%, 50%, 60%, and 70%, respectively. All the preparation is conducted as above for the SEM observation.

For the characterization of surface morphology of the 3D scaffold, the scaffolds were fabricated using the aforementioned 3D printing parameters and subsequently freeze-dried for a minimum of 6 h to produce the scaffolds. All the preparation is conducted as above for the SEM observation.

#### EDS analysis

In this study, EDS analysis was employed to identify and quantify the presence of carbon (C), oxygen (O), calcium (Ca), and phosphorus (P) elements within the hydrogel samples. The procedure followed was akin to that described for cross-sectional surface morphology testing. Briefly, the lyophilized scaffolds were sectioned and coated with a thin layer of gold using a Smart Coater (JEOL, Japan). They were then examined under a JSMIT100 microscope (JEOL, Japan) at an acceleration voltage of 15 kV. Mapping images were utilized to record the presence of the aforementioned elements in each sample, and the quantity of each element at 4 random points was detected, measured, and calculated.

#### Compression test

The compressive strength of NOCC-OXG and NOCC-OXG-BCP hydrogels was conducted according to a previous study with a few modifications [17]. In brief, cylindrical hydrogel samples with a diameter of 1 cm and a height of 1 cm were prepared and gelled at room temperature. After that, the experiment was performed by using the Texture Analyzer (TA.Xtplus, Stable Micro Systems, UK) in unconfined compression up to 50% strain at room temperature, where the probe diameter used for the compression testing is 35 mm (P/35). The experiment was performed in triplicate.

For the compression of 3D NOCC-OXG and NOCC-OXG-BCP scaffolds, scaffolds were printed with a height of 1 cm to yield the scaffolds. After that, the scaffolds were sliced to prepare the sample with the area of 1 ×1 cm^2^ and the compression test was conducted as above.

#### Porosity

At first, the volume of freeze-dried scaffolds’ geometry (*V*_s_) was calculated from their height and diameter. Next, each scaffold was initially weighted (*W*_o_) and then immersed in absolute ethanol overnight at room temperature. After that, the samples were taken out of ethanol, blotted to remove ethanol on the surface, and weighed immediately (*W*_e_). Volumes of the pores (*V*_p_) are calculated as follows:

$$V_{p}=\frac{W_{e}-W_{o}}{p_{e}}$$ (1)

where *p*_e_ is ethanol density at room temperature (*p*_e_ = 789 mg/ml). Finally, the porosity of each scaffold sample was calculated as follows:

$$\text{Porosity}\;\left(\%\right)=\frac{V_{p}}{V_{s}}\times 100\%$$ (2)

#### Swelling degree measurement

The extent of swelling of hydrated scaffold samples was assessed through gravimetric measurement. Initially, scaffold samples measuring 1 × 1 × 1 cm^3^ were prepared, and their initial weights were recorded. These samples were then placed into custom-made supporting holders equipped with a mesh bottom, with each holder positioned in a separate well of a 6-well plate. Subsequently, 10 ml of PBS solution (1×, pH 7.4) was added to each well to fully immerse the hydrogels. All samples were then incubated at 37 °C. At specific intervals, each sample was carefully removed from the PBS solution and weighed. The swelling degree was calculated using the following equation:

$$\text{Swelling degree}\;\left(\%\right)=\frac{W_{t}-W_{o}}{W_{o}}\times 100\%$$ (3)

where *W*_o_ and *W*_t_ are the initial weight and the weight at time *t* of the hydrogel samples, respectively.

#### In vitro degradation evaluation

Similar to the swelling test, the degradation of hydrogel samples was evaluated gravimetrically under simulated physiological conditions using PBS (1×, pH 7.4) solution and incubation at 37 °C. The initial weight of each sample was recorded as *W*_o_, and at specified intervals, the samples were weighed and denoted as *W*_t_. The experiment was conducted in triplicate and continued for a duration of 120 d. The remaining amount of each hydrogel sample *W*_d_ (%) at each time point was calculated by the following equation:

$$W_{d}\;\left(\%\right)=\frac{W_{t}}{W_{o}}\times 100\%$$ (4)

where *W*_o_ and *W*_t_ are the initial weight and the weight at time *t* of the hydrogel samples, respectively.

### In vitro testing

#### Cell culture

Cryotubes containing L929 mouse fibroblasts and rabbit bone marrow (R-BM) cells were thawed, and the cells were subsequently cultured in T-75 flasks using DMEM supplemented with 10% FBS and 1% antibiotics (penicillin and streptomycin) until they reached a confluency of 90%. Upon reaching this confluency, the cells were harvested using trypsin/EDTA 0.25%. Cell numbers were counted using a hemocytometer, and the cells were then seeded into a 96-well culture plate at a density of 10^4^ cells/well.

#### Live/dead assay

The in vitro biocompatibility of hydrogel samples was assessed using L929 mouse fibroblasts. Initially, hydrogel samples with a thickness of 1 mm were prepared in a 24-well culture plate and then freeze-dried and sterilized for the live/dead assay. A suspension of L929 fibroblasts was seeded onto the surface of the scaffolds at a density of 10^4^ cells per well in 100 μl of the culture medium and then incubated for 24 h at 37 °C. Subsequently, the culture media were removed, and the samples were washed with PBS solution. A working solution was prepared by adding 8 μl of fluorescein diacetate (FDA) (5 mg/ml) and 50 μl of propidium iodide (PI) (2 mg/ml) into every 5 ml of PBS to stain viable and dead cells, respectively. The working solution (200 μl) was added to each well, followed by 15 min of incubation, and then washed with PBS again. Finally, live and dead cells, visualized as green and red, respectively, on the scaffold samples were detected using a fluorescent microscope (Nikon, Japan). The experiment was conducted in triplicate.

#### In vitro cytotoxicity

The cytotoxicity of the hydrogels was evaluated using the Resazurin assay in accordance with the ISO 10993-5 Standard Test. Initially, 0.1 g/ml extracts of the hydrogels were prepared by incubating lyophilized samples in the cell culture medium at 37 °C for 24 h to obtain extracted solutions. These hydrogel extracts were then diluted to various concentrations, including 100%, 50%, and 25%. DMEM served as a control (0% extracted solution). Meanwhile, L929 fibroblasts were seeded into a 96-well culture plate at a density of 10^4^ cells per well in 100 μl of the culture medium and incubated at 37 °C for 24 h. Subsequently, the culture medium in each well was replaced with 100 μl of hydrogel extracts and further incubated at 37 °C for another 24 h. Following this, the hydrogel extracts in each well were substituted with 100 μl of FBS-free DMEM containing Resazurin 1× in the culture medium, and the plate was incubated at 37 °C for an additional 4 h. Finally, the fluorescent signal was measured at 530-nm excitation and 590-nm emission wavelengths using a Varioskan multiplate reader. The results were expressed as the percentage of computed cell viability compared to untreated control cells. The experiment was conducted in sextuplicate.

#### In vitro proliferation rate evaluation

The proliferation assessment of both the hydrogel extraction and the 3D-printed scaffold was performed simultaneously. For the hydrogel extraction, the procedure was conducted as previously described. Cells seeded in a 96-well plate were cultured at a concentration equivalent to 100% hydrogel extraction. Resazurin 1× was used to assess cell viability after 1, 3, 5, and 7 d, following the same method described earlier.

Regarding the 3D-printed scaffold, the scaffold was prepared as previously described and sterilized using the ethylene oxide (EO) method. Cell culturing on the 3D scaffold was carried out over 1, 3, 5, and 7 d. Once the cells reached a confluency of 90%, L929 fibroblasts were seeded onto the scaffold at a density of 10^3^ cells and incubated for determined intervals. The medium was changed every 2 d. The cell percentage was then measured using a microplate reader to detect fluorescent signals. Results were normalized to the cell percentage on day 1 and calculated to evaluate the proliferation ability of L929 cells on the 3D-printed scaffold.

#### L929 and R-BM cell attachment evaluation on scaffold’s surface

The 3D-printed scaffold is prepared as described above and sterilized by the EO method. The R-BM and L929 cell seeding method on the scaffold is conducted similar to the proliferation test on the 3D scaffold. After 7 d, all scaffolds are taken out and immersed in 2% glutaraldehyde overnight at 4 °C. Then, the scaffold was washed with PBS for 10 min at room temperature twice and then the water dehydration by ethanol was conducted at concentrations of 30%, 50%, 70%, 90%, and 100%, respectively. The scaffold was put in the holder full of 100% ethanol, and the process was started with a critical point dryer machine (Autosamdri-815, Series A, Tousimis). The scaffold was then taken out, and cell adhesion on the scaffold was observed with a JSMIT100 microscope (JEOL, Japan) at 5 to 10 kV.

#### Characterization of R-BM by multilineage differentiation

After reaching a confluency of approximately 80%, R-BM cells were detached with 0.25% trypsin/EDTA and centrifuged at 2,000 rpm for 5 min to collect cell residue. The cell pellet was then suspended into the DMEM culture medium, 10% FBS, and 1% antibiotics and seeded into a 96-well plate with a density of 1 × 10^4^ cells/200 μl of medium/well.

After 24 h, when cells had spread, the medium was replaced with the adipogenic, osteogenic, and chondrogenic differentiation medium for the corresponding differentiation wells. The medium was replaced every 3 d. After 21 d, the induction medium was removed and washed twice with PBS. Separately, the bones were not washed with PBS but instead with sterile autoclaved double-distilled water. Staining solutions including Oil Red O, Safranin O, and Alizarin Red S were used to correspond to cells that are induced to differentiate into fat, cartilage, and bone to detect the differentiation. After 10 min, the dye was removed and the excess dye was washed with PBS for cartilage and adipose differentiation, or distilled water for bone differentiation until the solution becomes clear. Images were recorded using an inverted microscope.

#### Detection of chondrogenic and osteogenic differentiation capacity of R-BMs under culturing of 3D scaffold extraction

After cell was cultured in 96-well-plate cell culturing, the culture medium was prepared based on the differentiated induction medium and scaffold extraction. In detail, the corresponding differentiated induction medium was mixed with the 100% scaffold extraction with a volume ratio of 1:1. Then, the culture medium was replaced once cell confluency reached approximately 80% corresponding to types of biomaterials nominated before for bone and chondrocyte regeneration. The differentiated induction medium was replaced every 3 d and observed for the change during the process. After 21 d, all the medium was discarded and proceeded to the staining process of Alizarin Red S for osteogenic differentiation detection and Safranin O for chondrogenic differentiation detection.

### In vivo testing

#### Scaffold for implant

In this experiment, the number of rabbit models utilized was 2 per sample group and timeline. Both knee joints of each rabbit were employed to generate the defect, resulting in a total of 4 lesions per sample group at each timeline. An osteochondral defect (3 mm in depth and 3 mm in diameter) was created in the center of the trochlear groove of the knee joint. The bilayered 3D-printed scaffolds were specifically designed and fabricated to conform to the defects created in a rabbit model as mentioned above. After lyophilization, the scaffolds were sectioned into cylindrical shapes measuring 3 mm in thickness and 3 mm in diameter. Particularly, the subchondral bone and cartilage layers are 2 and 1 mm in thickness, respectively. The scaffolds were sterilized with EO and then immersed in physiological saline and prepared for the implantation.

#### Rabbit osteochondral defect model

In vivo experiments adhered to the guidelines set by the Institutional Animal Care and Use Committee of Can Tho University of Medicine and Pharmacy (No. 24.002/PCT-HĐĐĐTĐV). New Zealand rabbits, each weighing approximately 3 kg, were anesthetized via intramuscular injection of 5 mg/kg xylazine (Rompun, Bayer) and 15 mg/kg Zoletil 50 (Virbac Co., France), and the fur around the leg area was shaved. All the equipment during the surgery and drilling is autoclaved and ensured to be sterilized throughout the process. Both knee joints of each rabbit were employed to generate the defect. The knee was bent slightly to identify the location of the tendon, and then the incision was created at about 5 mm medial to the tendon center to avoid the tendon damage. A longitudinal (parallel to the tendon) incision was made on the skin layer. A deeper incision was made at the same location to expose the fascia, which is thin and translucent, followed by the joint capsule, which is thicker and opaque. The incision should be stopped immediately when the knee is exposed to prevent bone damage. Forceps are used to retract the surrounding tissue out of the drilling site. The angle of the joint flexion was adjusted for easier access to the trochlear groove. An osteochondral defect (3 mm in depth and 3 mm in diameter) was created in the center of the trochlear groove of the knee joint using a trephine and a drill bit with continuous saline irrigation to rinse the whole process to remove debris and avoid local overheating. The debris from the interior is removed completely before the scaffold implantation.

The 3D-printed bilayered scaffolds were specifically designed to conform to the defects created in a rabbit model. The 3D scaffold preparation for implantation was conducted as described above. For osteochondral defect repair, NOCC-OXG-BCP hydrogel was used to create the bone tissue layer, with NOCC-OXG hydrogel layered on top to form the cartilage tissue layer.

The wound closure process proceeds within the reverse order of the knee incision procedure. The joint capsule was sutured first, followed by the fascia layer and skin layer. All the steps have to be conducted carefully to avoid damage to other structures of the knee and ensure the movement ability of the rabbit after the implantation process [20]. The rabbits were housed and monitored for recovery over periods of 4 and 8 weeks.

In the case of OAT, trephine with a diameter of 3 mm will be used to create the defect. As a result, the boundary between the trochlear groove and the surrounding area was removed, creating the OAT. The wound was sutured as described, and analyses were conducted in weeks 4 and 8.

#### X-ray analysis

The animals were sacrificed to evaluate the tissue regeneration in osteochondral defect by x-ray, at weeks 4 and 8 post-surgery. The harvested samples were stored in 10% (v/v) formalin, and then pictures were taken by computed tomography (CT) scan PaX-i3D Smart (PHT-30LFO).

#### Histological examination

After tissue collection, tissue staining is performed to evaluate the process of articular cartilage regeneration. Briefly, after the implantation, rabbits were sacrificed by intravenous injection of 150 mg/kg KCl after anesthesia with the same dose as presented in the above section. Osteochondral tissue was collected, and decalcification was performed in 5% nitric acid. The tissue was then soaked in 10% formalin, embedded in paraffin, sectioned into 5-μm thin slices, and stained with hematoxylin and eosin (H&E), Masson’s trichrome, and Safranin O/fast green.

### Statistical analysis

All experiments were performed in at least triplicate. Student’s *t* test and one-way analysis of variance (ANOVA) were used to find significant differences between groups (Tukey post hoc test; SPSS 20 software, IBM Corp.), and a statistically significant difference was defined as *P* < 0.05. The data were given in the form of a mean and standard deviation (SD).

## Results and Discussion

**Fig. 2.**
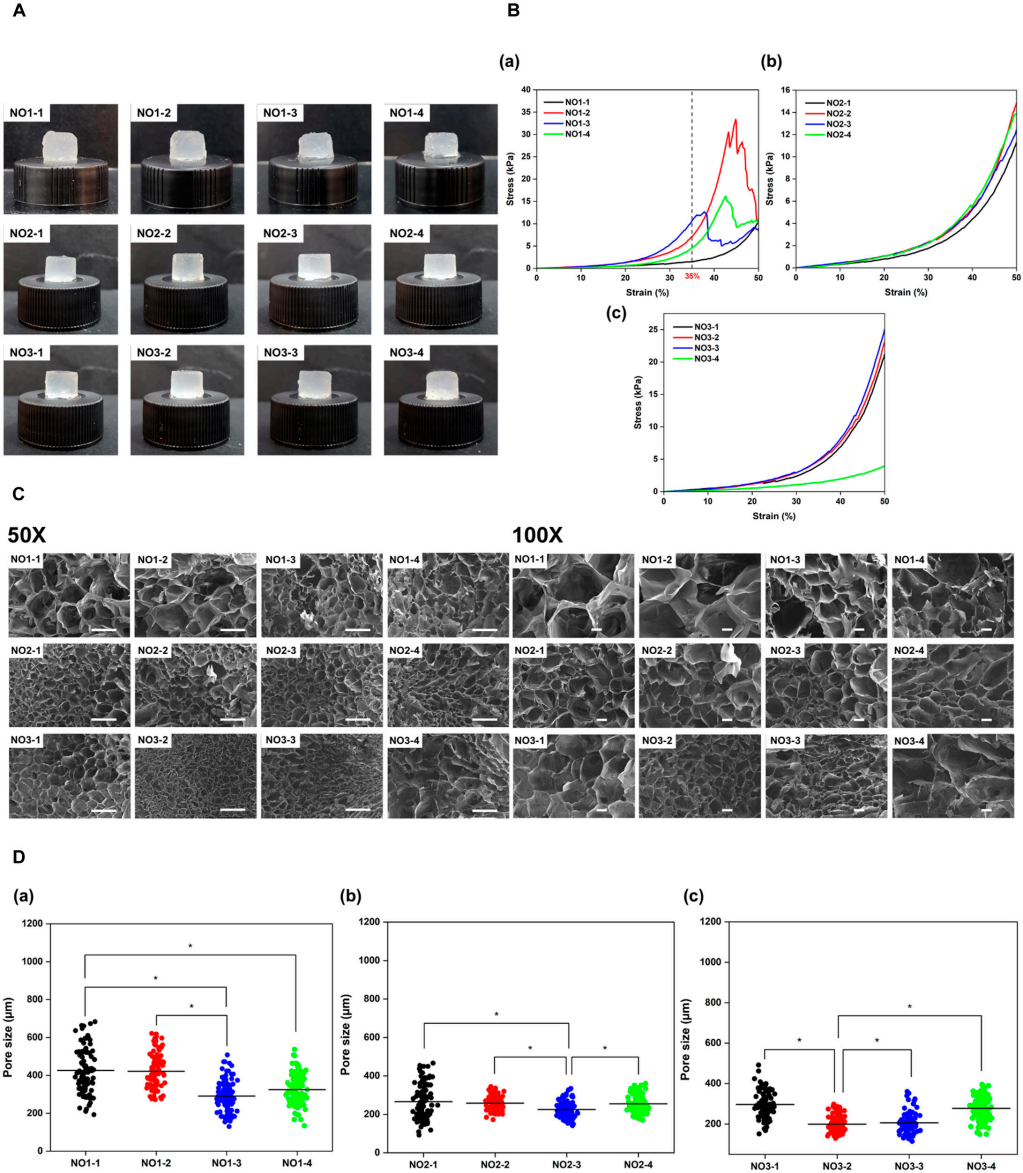
Characterization of NOCC-OXG hydrogel sample at different oxidation levels of OXG and various volume ratios between NOCC and OXG. (A) Hydrogel images. (B) Stress and strain curve at the strain from 0% to 50% of (a) NO1, (b) NO2, and (c) NO3 hydrogel group. (C) SEM morphology images of NOCC-OXG hydrogel sample at different oxidations of OXG and volume ratios between NOCC-OXG at the magnification of 50× (scale bar, 500 μm) and 100× (scale bar, 100 μm). (D) Pore size distribution of NOCC-OXG hydrogel samples.

First, the volume ratio between NOCC:OXG and the oxidation level of OXG were optimized toward the most appropriate physical properties when compared to the native structure of cartilage tissue, through the experiments of the cross-sectional structure and compression strength under a certain bearing capacity, as shown in Fig. 2. As can be seen in Fig. 2A, the gel formation of different hydrogel sample groups showed no marked difference in their appearance, except the NO1 hydrogel group. While NO1 samples exhibited a slightly softer structure, NO2 and NO3 samples posed a firmer appearance within lesser gel formation time.

The compressive strength was measured to reveal the influences of various conditions on the force-bearing capacity of hydrogel samples. Figure 2B shows the stress and strain curve of the NOCC-OXG hydrogel sample at the gel state observed under the strain from 0% to 50%. In general, the higher oxidation level of OXG contributed to the higher compressive strength of the hydrogel sample. The NO1 hydrogel sample exhibited the breakpoint at the strain of 35% with the stress at approximately 10 kPa, while NO2 and NO3 hydrogels could maintain the state of the original hydrogel block up to 50% strain with a higher value of compressive strengths. In detail, all volume ratios of the NO2 hydrogel sample posed the same bearing capacity trends with the stress of approximately 12 to 14 kPa, increased to 25 kPa recorded at the similar strain of the NO3 hydrogel sample.

The inner structure of lyophilized samples was investigated through the cross-sectional surface morphology, as shown in Fig. 2C. In general, all the samples formed a hydrogel block with a complete porous structure. The NO1 group showed the largest pore size of approximately 430 ± 123.62 μm with the thinnest pore wall. In contrast, 2 other groups exhibited a smaller pore size with thick, firm pore walls, recorded at 260.60 ± 39.02 μm and 199.22 ± 44.28 μm for the NO2 and NO3 groups, respectively (Fig. 2D). When the OXG oxidation level increased, the pore structure seemed to be damaged and appeared nonuniformity. This might be due to the high viscosity of the OXG3, which made it difficult to equally mix the solution of NOCC and OXG to create the complete hydrogel without damaging its inner structure.

For cartilage tissue regeneration, a previous study found that collagen scaffolds with pore sizes ranging from 50 to 300 μm are typically favorable for stimulating the production of cartilaginous tissue, which is in line with another research that found that chondrogenesis was increased by collagen–HA scaffolds with pore diameters ranging from 90 to 300 μm [21]. From the experiment above, NO2 samples exhibited lower force-bearing capacity than NO3 groups at the similar strain but posing a more uniformly porous structure within the undamaged structure, which may be favorable for the biomaterial–cellular interaction toward the enhancement in the tissue regeneration process. Among NO2 hydrogels, NO2-2 exhibited the significantly highest force-bearing capacity as well as appropriate pore size and distribution. Therefore, NO2-2, abbreviated as NO2 in further experiment, would be chosen as the appropriate candidate for the BCP incorporation of subchondral bone layer regeneration.

**Fig. 3.**
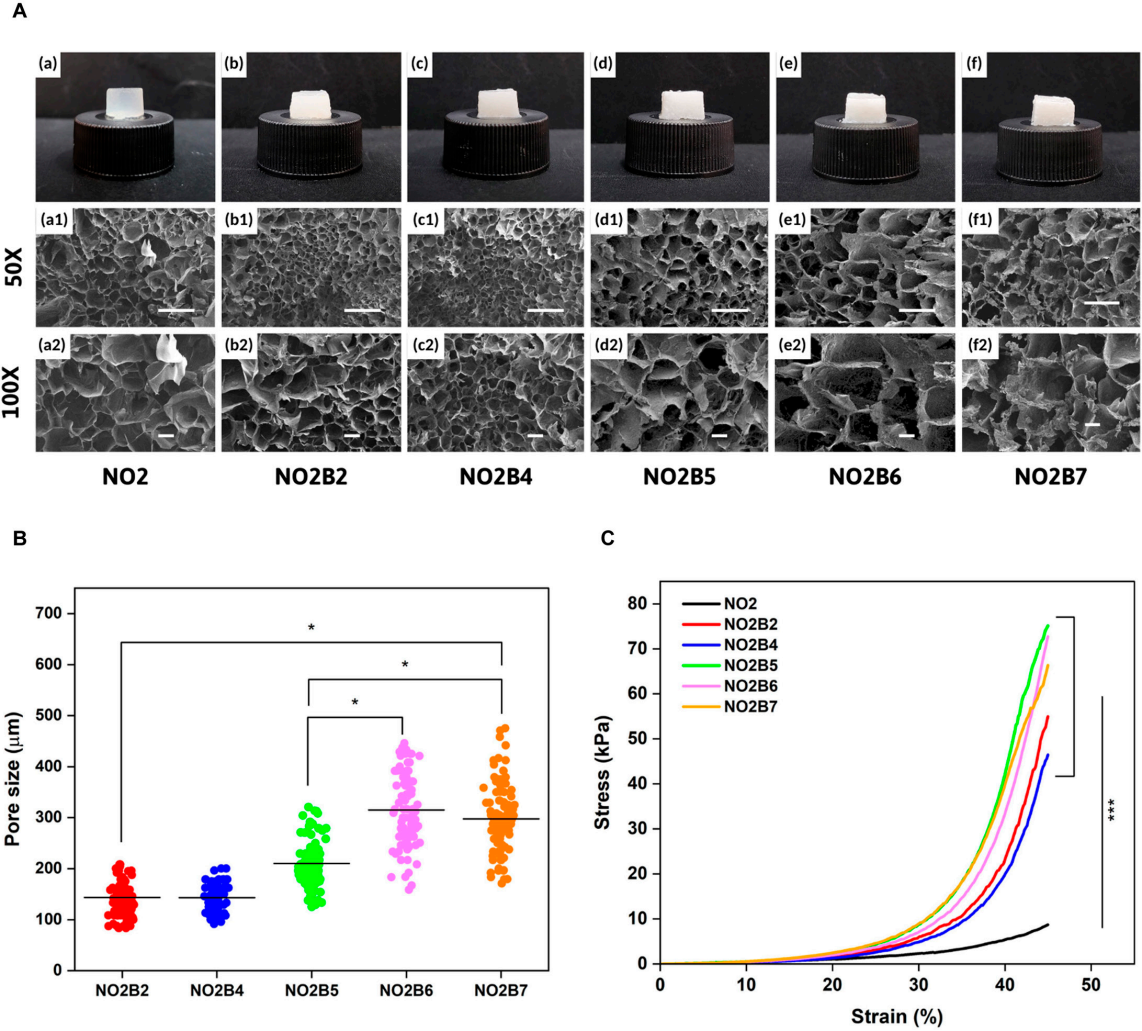
Characterization of NOCC-OXG-BCP hydrogel samples under various contents of BCP powder. (A) NOCC-OXG-BCP hydrogel sample gel image and SEM morphology images of NOCC-OXG-BCP hydrogel sample with variation of BCP powder (20%, 40%, 50% 60%, and 70%) at the magnification of 50× (scale bar, 500 μm) and 100× (scale bar, 100 μm). (B) Pore size distribution of hydrogel samples. (C) Stress and strain curve of hydrogel samples at the gel state at the strain from 0% to 45%.

To fabricate the osteoconductive-support scaffold, the BCP powder at different ratios was incorporated into the existing NOCC-OXG hydrogel with the purpose of optimizing the BCP content based on the native characteristics of the subchondral bone layer. Similar to NOCC-OXG hydrogel samples, gel image and SEM morphology within compressive strength were demonstrated to evaluate the physical properties of the scaffolds. Apparently, in Fig. 3A, the milky appearance of NO2B gel samples stated the successful incorporation of the BCP powder into the NOCC-OXG hydrogel sample when compared to the transparent color of the NO2 sample. Through the cross-sectional surface morphology, rougher appearance and thicker pore wall were revealed in the NO2B hydrogel group. At the low ratio of the BCP powder, NO2B2 and NO2B4 groups demonstrated the smaller pore size with nonuniformity, at an average of 145 μm, while less interconnectivity was observed throughout their structure. Once a higher ratio of the BCP powder was applied, their pore sizes increased from 210.37 ± 45.95 μm to 315.14 ± 76.47 μm (Fig. 3B) with varying interconnectivity, and thicker pore walls were demonstrated through 100× magnification of the SEM image.

The compressive strength of the scaffold was illustrated in Fig. 3C, presenting the effect of different ratios of the BCP powder on the mechanical properties of the hydrogel systems. Without the BCP content, significantly lower mechanical strength was recorded at the NO2 sample, 8.71 ± 0.29 kPa particularly, while the NO2B hydrogel posed the higher force-bearing capacity, ranging from 46 to 75 kPa. NO2B2 and NO2B4 showed significantly lower compressive strength when compared to the other BCP-loading groups, particularly exhibiting 54.97 ± 1.83 kPa and 46.46 ± 1.63 kPa, respectively. Three other samples with 50%, 60%, and 70% BCP content presented higher values but not significant differences among them. To be more detailed, NO2B5 showed the highest compressive strength at 75.13 ± 1.51 kPa, followed by 72.72 ± 2.77 kPa of the NO2B6 hydrogel group and 66.32 ± 1.94 kPa of the NO2B7 hydrogel group. By posing larger pore sizes from 200 to 300 μm, NO2B5, NO2B6, and NO2B7 showed better support for cell survival and proliferation [22]. Furthermore, the combination of NOCC and BCP together stiffened the matrix by a strong coordination bond between the NH_2_ of NOCC and Ca^2+^ of BCP, resulting in higher mechanical properties of the NO2B hydrogel sample [23]. Therefore, through cross-sectional image, pore size distribution, and compression strength evaluation, NO2B2 and NO2B4 groups have been excluded from the sample test for further experiments.

**Fig. 4.**
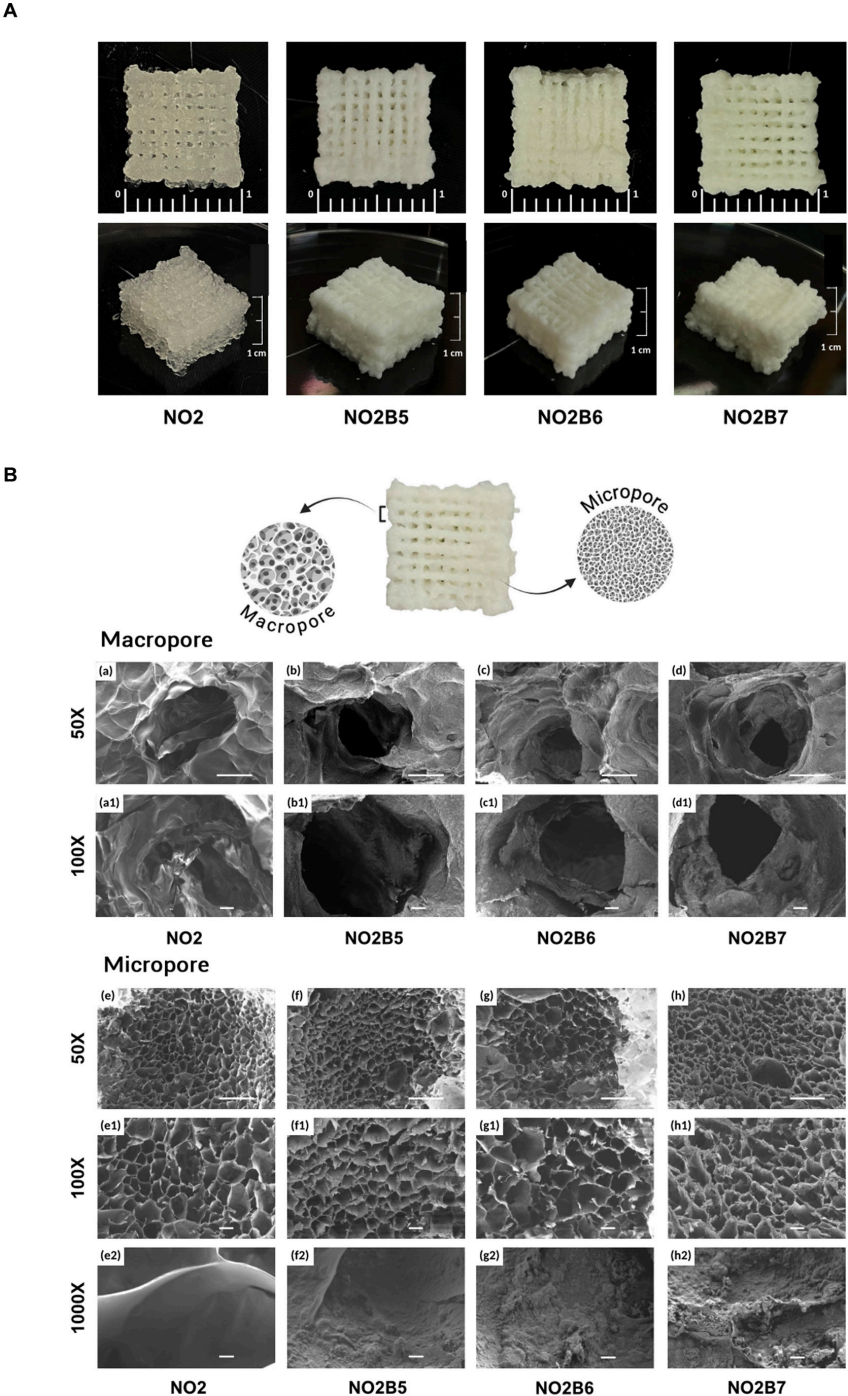
(A) 3D-printed 1 × 1 × 1 cm^3^ cube scaffold images of NO2 and NO2B hydrogel, and (B) SEM images of 3D-printed scaffold including macropore of NO2 (a and a1), NO2B5 (b and b1), NO2B6 (c and c1), and NO2B7 (d and d1) at magnification of 50× and 100× (scale bars, 500 and 100 μm) and micropore of NO2 (e, e1, and e2), NO2B5 (f, f1, and f2), NO2B6 (g, g1, and g2), and NO2B7 (h, h1, and h2) at magnification of 50×, 100×, and 1,000× (scale bars, 500, 100, and 10 μm).

Once the ratios were optimized, a 3D printing experiment was performed to assess the printability of each scaffold type under varying parameters. Pressure, printing speed, scaffold design, and other parameters were investigated for the successful 3D printing scaffolds. After the modification process, the 3D scaffold was printed at a pressure ranging from 1 to 3 MPa, G22 nozzle tip, and printing speed of 650 mm/min to allow the continuous flow of hydrogel solution. With the zigzag shape, changing the printing angle between layers to 90^o^ with an infill level of 35%, macropore structures would be formed throughout the printed structure. This design would be supportive of the vascularization through the macropore during the printing process, while the micropore structure was still expressed on the strands of the printed line. Scaffolds with the dimension of 1 × 1 × 1 cm^3^ were successfully printed, as shown in Fig. 4A. Visually, all scaffolds showed rigid and porous structures. However, when a higher amount of the BCP content was applied into the systems, it resulted in a dryer status with the same time duration, which led to the difficulty in controlling the continuous flow of the hydrogel out of the needle without clogging.

Cross-sectional surface morphology of the scaffold was conducted by SEM technique to analyze their inner structure. Theoretically, 3D printing brings the massive advantage of providing a wide range of macroporosity along with microporosity, which serves for both nutrients, oxygen exchange, and vascularization to enhance the efficacy of bone tissue regeneration. As can be seen from Fig. 4B, the macropore was created by the infill of the design, particularly 35% infill over 1-cm length. Generally, all scaffold samples exhibited the macropore ranging from 600 μm to approximately 1,500 μm, with NO2’s macropore recorded as the smallest size when compared to other groups. This may be due to the little BCP content existing in the hydrogel system, resulting in a considerable decrease in crosslinking of the hydrogel, thus leading to the rupture of hydrogel flow and reducing the macropore size. Meanwhile, the incorporation of the BCP powder into the hydrogel systems made it gel faster with a more rigid structure, thus improving the stability of the gel. As a result, large macropores were exhibited at all 3 scaffold groups: 1,393.01 ± 251.04 μm of NO2B5, 1,437.24 ± 195.73 μm of NO2B6, and 1,412.28 ± 184.42 μm of NO2B7. In terms of the macropore of the 3D scaffold, the macropore ranging from 600 to 1,500 μm was suitable for the vascularization process, which plays the core role of bone implantation [24]. After optimizing the infill of 35% over the length of the scaffold, it is evaluated as suitable to maintain the large pore size for the BCP-containing hydrogel to facilitate the neo-angiogenesis process, while resulting in the disappearance of the large macropore throughout the NOCC-OXG layers suitable for the native chondrocyte layer along with the nonvascularization characteristics [25].

The micropore of the 3D printing scaffold was subsequently investigated. As have been mentioned above, no significant difference in their average pore size was demonstrated among NO2 and NO2B scaffold samples. Although a slight increase in average pore size from NO2, NO2B5, and NO2B6 groups was detected, particularly 135.85 ± 38.86, 172.83 ± 49.93, and 249.82 ± 85.63 μm, respectively, the pore size exhibited a drop to 186.14 ± 82.17 μm when the BCP content reached 70%. No influence was caused to affect the cell–biomaterial interaction such as cell adhesion and proliferation, as the value from 135 to 250 μm was suitable for cell attachment, migration, proliferation [24]. Thus, the 3D-printed scaffolds’ inner structure was evaluated as qualified for bone–cartilage regeneration.

**Fig. 5.**
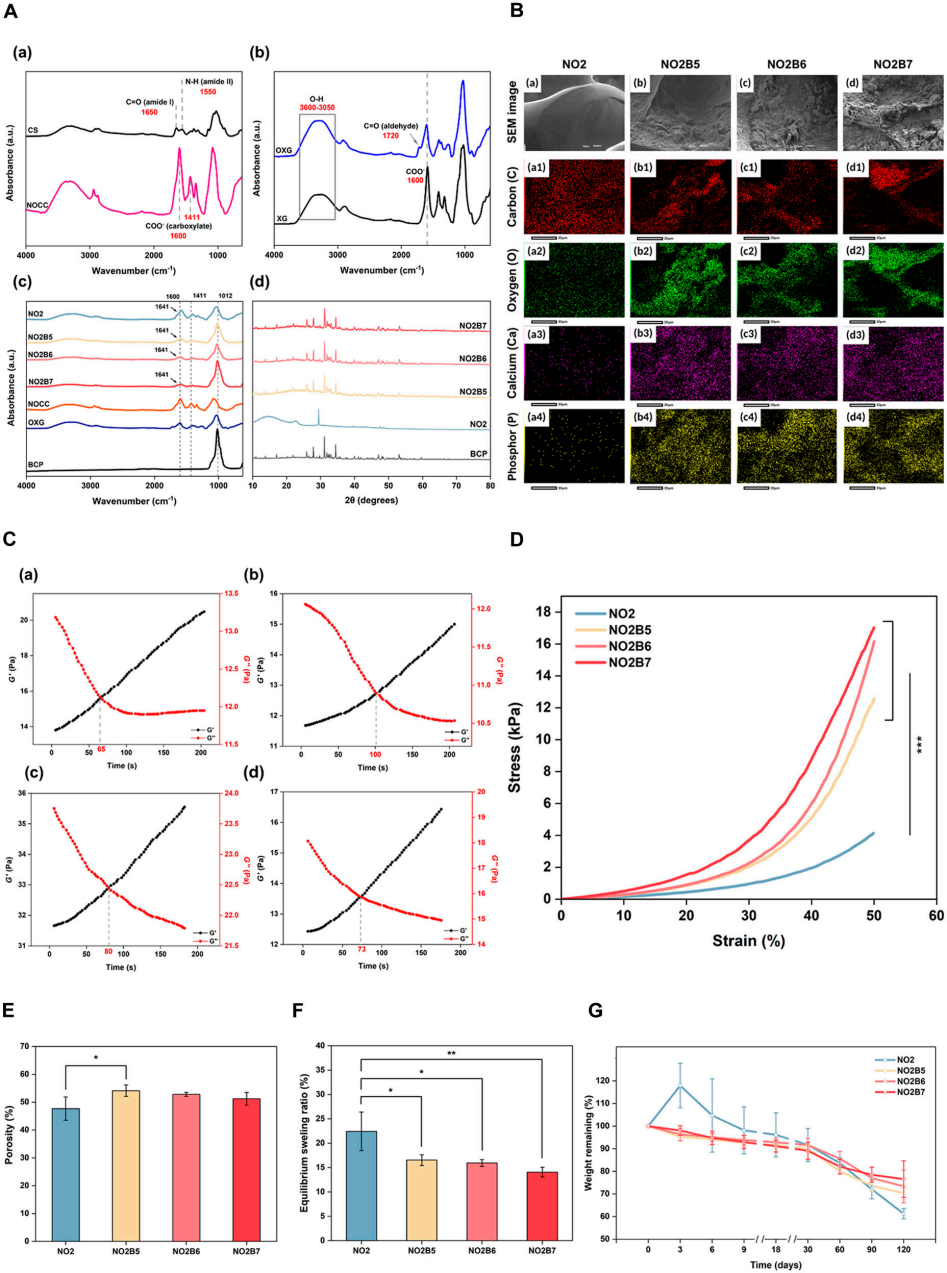
Characterization of NOCC-OXG-BCP 3D-printed scaffold. (A) FTIR spectra of (a) CS and NOCC, (b) XG and OXG, and (c) NOCC, OXG, BCP, NO2, and NO2B hydrogels at 3 different BCP contents and (d) XRD analysis of NO2 and NO2B hydrogel samples and BCP powder. (B) EDS mapping images of NO2 (a, a1, a2, a3, and a4), NO2B5 (b, b1, b2, b3, and b4), NO2B6 (c, c1, c2, c3, and c4), and NO2B7 (d, d1, d2, d3, and d4) scaffold samples at 1,000× magnification (scale bar, 30 μm). (C) Gelation time of (a) NO2, (b) NO2B5, (c) NO2B6, and (d) NO2B7 hydrogel samples. (D) Stress and strain curve of NO2 and NO2B scaffold sample at the hydrated state at 50% strain. (E) Porosity. (F) Equilibrium swelling degree obtained at 3.5 h of being immersed in PBS solution at 37 °C. (G) In vitro degradation assessment of NO2 and NO2B scaffold samples. *P < 0.05; **P < 0.01; ***P < 0.001.

FTIR spectra of NOCC, OXG, and hydrogel samples were analyzed and shown in Fig. 5A. For CS, absorption peaks of C–O stretching, N–H bending of N-acetyl groups were observed at 1,650 and 1,550 cm^−1^, respectively. By grafting the carboxylate group, the emerging strong, broad bands at 1,600 and 1,411 cm^−1^ were attributed to -COO^−^ asymmetric and symmetric stretching vibration, confirming the successful introduction of these chemical groups into CS modification into NOCC [16]. For OXG, the spectra of OXG mostly show the same as that on XG. However, the existence of 1,720 cm^−1^ peak marked the introduction of the aldehyde group into the modification of XG, representing C=O stretching vibration of the aldehyde group on OXG’s spectra [18]. Theoretically, the anticipated C=N linkage peaks were detectable at 1,641 cm^−1^, which is representative of the Schiff base crosslinking formation; however, the peak was hardly seen due to the overlapping with the adjacent stronger and broader -COO- stretching peak of carboxymethyl groups. Moreover, the large peak at 1,012 cm^−1^ of BCP’s spectra was also reflected in 3 groups of BCP-incorporated hydrogel samples, confirming the existence of the BCP content in the structure of the hydrogel systems when compared to the NO2 group [17].

On account of incorporating the BCP powder into the hydrogel system, the crystalline nature of the BCP powder was detected through XRD analysis with the purpose of proving the successful incorporation of the BCP content. At the same time, the EDS method was simultaneously conducted to investigate the elemental component analysis of the scaffold under the circumstances of different ratios of the BCP content, as shown in Fig. 5A and B. Consisting of β-TCP and HAp composition, peaks of both components were analyzed on the graph within the appearance of a new peak of BCP. Particularly, the crystalline nature of the β-TCP sample was observed at 2θ of 25.8°, 29.6°, 32.4°, and 39.8°. Besides, HAp peaks were shown at 27.8°, 31°, and 34.3°, while the peak at 34.2° existed, which belonged to the BCP content. Obviously, the result illustrated the important differences between NO2 and NO2B hydrogel scaffolds. On the reflection pattern of all 3 scaffold groups with BCP incorporation, they all appeared at the peak of β-TCP, HAp, and BCP, while no peak detection of BCP was reflected on NO2’s pattern. These results have proved the success of BCP incorporation into the hydrogel system of all 3 groups in the mapping element evaluation. All constituent elements contributing to the formation of the hydrogel components were identified in all samples, with the signal intensity of these elements being notably high. Remarkably, the increase in the BCP content of the hydrogel system was demonstrated directly by the higher Ca and P components shown in Table S1. For the NO2 scaffold sample, very little amount of Ca and P was detected as the non-incorporation of the BCP powder into the hydrogel system occurred, regarded as Nd of the Ca and P element, while with scaffold systems with BCP incorporation, the Ca element was recorded at 20.07 to 25.55% and 12.22 to 15.35% for the P element.

The gelation time of NOCC-OXG and NOCC-OXG-BCP hydrogels was demonstrated in Fig. 5C to reveal the different gelation abilities under various conditions. In general, in all groups, hydrogels were formed at relatively short time, ranging from 60 to 100 s recorded. As can be seen, the NO2 hydrogel had the shortest gelation time, at about 65 ± 23.3 s. A combination of the BCP content in the hydrogel systems seemed to interrupt the crosslinking between NOCC and OXG, thus causing the phenomenon of longer gelation time, which was observed at NO2B hydrogel samples. Meanwhile, the higher ratio of the BCP content tended to add up more hydrogen bonds as well as coordination bonds between NH_2_ and Ca^2+^, therefore gradually decreasing the gelation time. Particularly, the gelation time of NO2B5 was recorded at 100 ± 14.6 s, while that of NO2B6 decreased to 80 ± 23.4 s and NO2B7 gelled at 73 ± 34.5 s. With the recorded gelation time, it was evaluated as appropriate for injectable hydrogel application that filled in various defect sites, including the 3D printing application [26].

The compressive strength of the 3D-printed scaffold within 1 × 1 × 1 cm^3^ parameters was evaluated and shown in Fig. 5D to examine the force-bearing capacity of the scaffold. Similar to the result of the hydrogel blocks, the NO2B 3D-printed scaffold exhibited superior mechanical properties under the same strain when compared to the non-BCP sample. At 50% strain, the NO2 scaffold exhibited 4.16 ± 1.09 kPa, while NO2B groups were recorded at higher value ranging from 12 to 17. To be more detailed, NO2B7 showed the highest compressive strength at 17.01 ± 4.08 kPa, followed by 16.16 ± 4.04 kPa of NO2B6 and 12.57 ± 3.26 kPa of the NO2B5 hydrogel group. These results indicated a significant increase in compressibility when BCP was incorporated into the polymer system, which was partially explained by ionic crosslinking between the divalent cation Ca^2+^ in BCP and the carboxylate groups in OXG within the strong coordination bond between the NH_2_ of NOCC and Ca^2+^ of BCP, indicating that the presence of BCP effectively increased the mechanical strength of the fabricated scaffolds [27]. In comparison between the force-bearing capacity of the hydrogel block and 3D scaffold, the decrease in compressive strength could be informed by the 35% infill over the total size of the scaffold, resulting in space between the printed strands. To reach the balance between compression ability and vascularization for regeneration purposes, this infill printing design has been given out to serve as the most effective approach for tissue regeneration.

The porosity of hydrogel structures and their swelling capacity play a vital role contributing to the mechanical and biological properties of the scaffold. After the immersion in PBS solution at 37 °C at different periods, porosity, swelling rate, and biodegradation rate of all scaffolds were detected, as shown in Fig. 5E to G. When increasing the incorporation of the BCP powder into the NO2 hydrogel sample, the decreased proportionality in both porosity and swelling rate existed. The porosity of the scaffold sample was recorded, with values of 54.15 ± 2.02%, 52.81 ± 0.7%, and 51.23 ± 2.28% for NO2B5, NO2B6, and NO2B7, respectively. The excessive accumulation of BCP particles on the scaffold surface, leading to reduced porosity, was caused by incomplete dissolution of BCP powder in the NOCC solution. This accumulation, as Amin et al. suggested, is likely driven by the release of ions from the BCP particles and their subsequent surface precipitation [28]. Proportional to the porosity of the scaffold, there was a decreasing trend in the swelling ratio by increasing the BCP content. In terms of the swelling rate of the scaffold, the appropriate rate may facilitate the absorption of nutrients from the surrounding liquid, creating a conducive environment for cell survival and growth, thus enhancing the scaffold’s bioactivity. As recorded, the hydrogel sample with a 50% BCP ratio exhibited the highest equilibrium swelling degree at 3.5 h at the value of 16.55 ± 1.12%. This gradually decreased to approximately 15.95 ± 0.71% and 14.05 ± 1.01% for the NO2B6 and NO2B7 samples, respectively. The swelling capacity primarily relied on the abundance of hydrophilic groups, which enhanced the copolymer’s affinity to water molecules, consequently leading to a higher swelling capacity, as observed in the NO2 sample. However, the incorporation of the BCP content might disrupt the bonding between NOCC, OXG, and water molecules, occupying the space that would otherwise be filled by water molecules [28]. This interference resulted in a reduced swelling degree in the NO2B scaffold groups. A decrease in swelling degree could potentially impact the exchange of nutrients crucial for cell survival, while a higher swelling degree, indicative of greater porosity, might lower the load-bearing capacity of the scaffold. Hence, it is essential to strike a balance by considering the average rates of porosity and swelling in the scaffold. Consequently, the porosity and swelling degree observed in the NO2B scaffold could be deemed suitable for facilitating nutrient exchange and cellular behavior while maintaining an average force-resistance capacity.

The biodegradability of polymeric hydrogels is another crucial factor that attributes in the context of tissue engineering, as these materials need to be broken down and eliminated from the implanted site once they have fulfilled their intended function. The rate of degradation must be carefully controlled to accommodate the formation of newly produced ECM while simultaneously preserving mechanical support [29]. Generally, all the 3D-printed scaffolds exhibited the downward weight remaining after 120 d as the provident of the degradation of the scaffold immersed in the PBS solution, recorded more than 60% of the total scaffold volume at day 120. After 4 months of immersion, NO2B groups maintained a larger gel volume than the NO2 scaffold sample. From the beginning of day 3, the NO2 sample showed an increase in weight due to the water element absorption into the scaffold structure. Then, all the scaffold groups started the degradation process and gradually decreased to more than 60% of the total weight at day 120. Particularly, the NO2 scaffold posed the least gel volume after 120 d of 61.29 ± 4.82%. This can be explained by the majority of the macromolecule of hydrophilic groups, the scaffold was hydrolyzed quickly due to the existence of water, leading to a faster degradation rate. Meanwhile, a larger content of BCP incorporated inside the scaffold system resulted in higher remained gel volume, recorded 70.34 ± 5.12% of NO2B5, 73.35 ± 10.34% of NO2B6, and 76.56 ± 13.43% of NO2B7. Through the interaction among NOCC, OXG, and BCP, a more stable, rigid, and compact scaffold structure appeared, leading to little reduction in the degradation rate [30]. Overall, with the higher porosity and swelling rate, the NO2 scaffold was considered to be appropriate for cartilage tissue regeneration. While gel volume was not significantly changeable and remained up to 50% after 3 months of NO2B groups, the addition of the BCP powder was able to contribute to support for the mechanical strength and provide a suitable degradation rate for bone tissue regeneration.

**Fig. 6.**
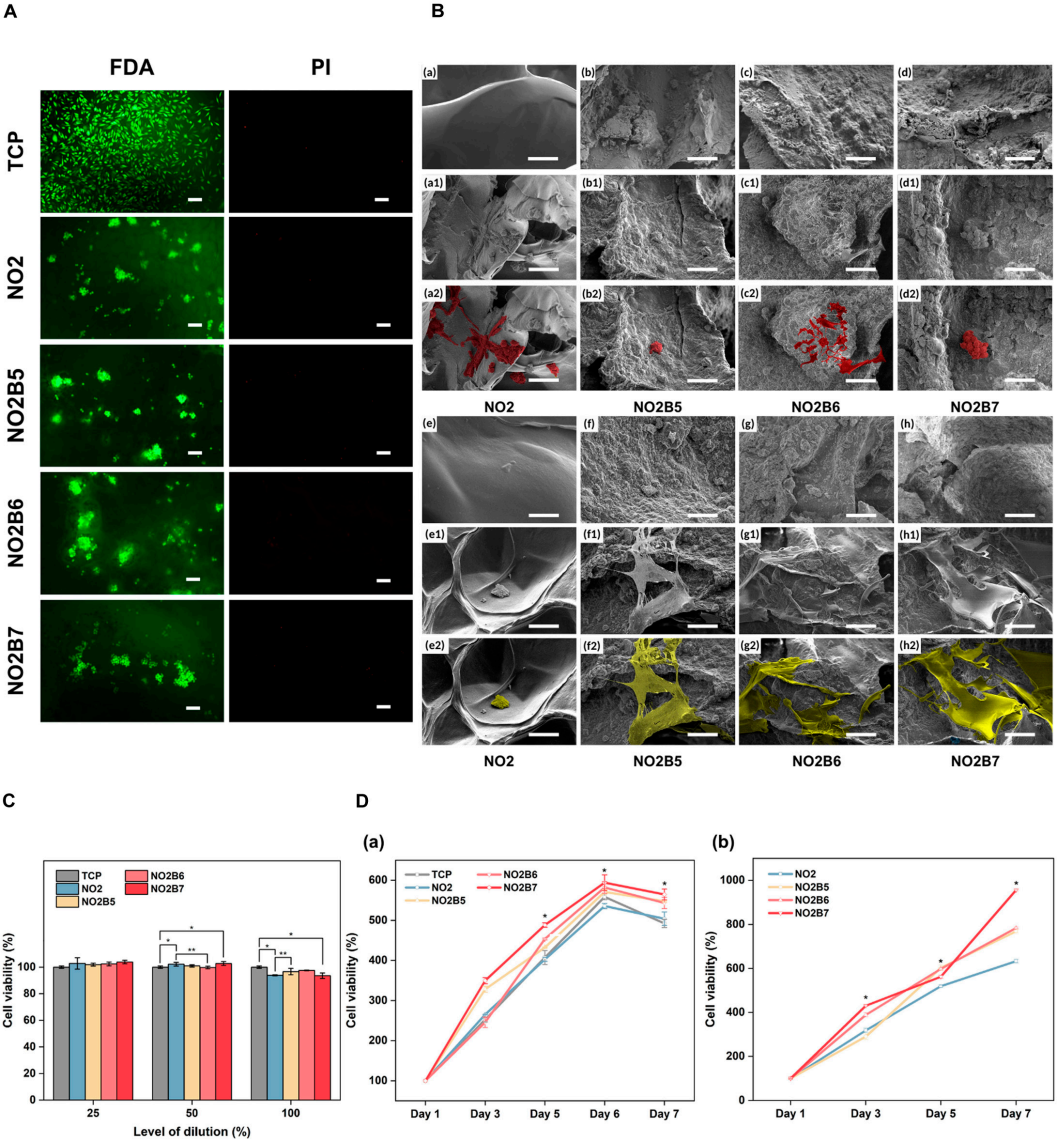
In vitro evaluation. (A) FDA/PI staining of L929 cells on TCP and NO2 with NO2B scaffold samples after 1-d culturing (scale bar, 100 μm). (B) Adhesion of L929 cells observed on the surface of 3D scaffold (a, b, c, and d: control without cells; a1, b1, c1, and d1: cells spreading on the surface; a2, b2, c2, and d2: highlighted cells on the surface in red color) and adhesion of R-BM cells observed on the surface of 3D scaffold (e, f, g, and h: control without cells; e1, f1, g1, and h1: cells spreading on the surface; e2, f2, g2, and h2: highlighted cells on the surface in yellow color) at magnification of 500× (scale bar, 50 μm). (C) Cytotoxicity evaluation of NO2 and NO2B scaffold samples using Resazurin assay (*P < 0.05, **P < 0.01). (D) Proliferation evaluation of L929 cells in (a) scaffold’s extraction and (b) 3D-printed scaffold for 1 week. (*: cell viability of NO2B5, NO2B6, and NO2B7 is significantly different with NO2).

In vitro studies were then conducted to test the biocompatibility and bioactivity of cells cultured with the 3D-printed scaffold. First, the live/dead assay was conducted to examine the cell survival and adhesion on the surface of the 3D scaffold, shown in Fig. 6A. By the FDA/PI label, FDA-labeled green fluorescence is demonstrated as live cells, and PI-labeled red fluorescence is seen as dead cells. Generally, all groups presented large number of green dots with very few red dots, which indicated that a few cells appeared dead after 1 d of culturing. While microscopic observation revealed high cell confluency in the tissue culture plate (TCP) group, the hydrogel samples facilitated cell growth at diverse altitudes, hindering the identification of individual cells. However, the superiority of live cells compared to dead cells of all groups revealed the high potential ability of the hydrogel to support cell survival, adhesion, and proliferation when compared to the TCP group.

To examine the adhesion ability of L929 and R-BM cells on the surface of the scaffold, SEM images for the cross-sectional observation were conducted. After culturing on the scaffold’s surface for 7 d, the scaffold with cell adhesion was collected, and critical point dryer was then applied to preserve the cell morphology as well as the scaffold’s shape and analyzed in Fig. 6B. Apparently, L929 cells exhibited the not completely spreading of their morphology on the culturing surface of experimental scaffolds, while R-BM cells presented the well-spread and adhesive properties on the BCP-supplemented groups, except on the NO2 sample. Specifically, NO2B6 demonstrated a good supportive capacity for cell adhesion and spreading for both L929 and R-BM cells, which is superior to other samples. It could be inferred that experimental scaffolds posed the unsuitable supplemented environment for the L929 cells to adhere and spread, but for R-BM cells. This result was aligned with the anticipated outcome of research to promote a more bone-like phenotype as R-BM cells for bone regeneration. Moreover, the differences among NO2 and NO2B groups emphasized the functioning of BCP supplementation into the system for the biomaterial–cellular behavior and interaction, consequence to the enhancement in regeneration efficiency.

Extraction of the scaffold was used to examine the biocompatibility of cell biomaterial. Hydrogel extraction is prepared by hydrophilizing the scaffold into the DMEM culture medium for 1 d to collect the hydrogel extraction. To check the influence of the hydrogel extraction with different concentrations, all the extraction (100%) is prepared and diluted to 50% and 25%, respectively. The Resazurin assay was used to evaluate the cytotoxicity of the 3D-printed scaffold on L929 mouse fibroblasts, while culture on the culture disk is noted as the standard condition for the control group. The cell viability rates of four 3D scaffolds within the control group are shown in Fig. 6C. Notably, the change in the BCP content and the extract concentrations among samples did not significantly affect the cell viability in this situation. It was calculated that all the scaffold samples recorded the cell viability of higher than 90% even at 100% extraction concentration, particularly, 93.86 ± 0.42%, 96.69 ± 2.42%, 97.59 ± 0.33%, and 93.59 ± 2.00% in NO2, NO2B5, NO2B6, and NO2B7, respectively. This concluded that scaffolds caused no cytotoxicity as ISO-1993-5-2009 stated, advantageous for the proliferation assessment to verify the capability of biocompatibility and bioactivity of the 3D scaffold.

After confirming that no cytotoxic effect was caused by the 3D scaffold, the cell proliferation ability culturing in both scaffold extraction and 3D-printed scaffold was conducted to examine whether they exhibited the capacity for inducing cell proliferation or not. The process was evaluated through 1, 3, 5, and 7 d, shown in Fig. 6D. In the scaffold extraction, all 4 groups with the control sample demonstrated the same trend of increasing due on day 6 and dropping on day 7. Significantly, the NO2 sample showed a lower proliferation capacity when compared to the other 3 groups with BCP incorporation into the hydrogel system, and higher cell viability along with a larger content of BCP incorporation into the hydrogel system existed. At day 6, all 4 groups with the control sample presented the highest peak of cell proliferation capacity, particularly 535.49 ± 6.54%, 570.78 ± 5.51%, 581.8 ± 14.52%, and 593.74 ± 19.6% of NO2, NO2B5, NO2B6, and NO2B7, respectively. However, cell viability started to reach confluency and entered the plateau phase with the apoptosis process, leading to gradually decreased cell number at day 7. On the contrary, the 3D-printed scaffold facilitated cell survival and proliferation through the continuous increase at day 7, which may reached up to 955.79 ± 3.23% NO2B7 scaffold sample. These results indicated the superior support of the 3D scaffold over its extraction, since the 3D scaffold provided more space for cells to grow and proliferate and more nutrients to exchange and differentiate. Better support for cell proliferation was recorded on NO2B scaffold groups, as the result of BCP powder incorporation. With the supplementation of BCP particles, surface roughness might be enhanced by the additional anchoring sites, leading to the enhancement on the cell–substrate interaction on the scaffold [31]. Furthermore, the topography of a surface could have a substantial impact on the protein adsorption onto substrates, hence facilitating the regeneration process. Xing et al. [32] also stated that the biological effect of BCP had an influence on the regulation of bioactive molecules and gene expression that supported the bone tissue regeneration. In particular, calcium ions released from the degradation process were able to contribute to the growth factor expression and support the cells’ skeleton to adhere to the scaffold, therefore enhancing the contact between cells, biomaterials, and surrounding microenvironment toward cell proliferation, differentiation, and mineralization [33].

Overall, the NO2 scaffold was evaluated as an appropriate natural-originated scaffold that closely mimicked the native ECM of cartilage tissue with optimized ratio for corresponding physical and biological properties such as porosity, swelling rate, and nontoxic bioproduct. Moreover, the positively charged state of NOCC allowed the interaction with the negatively charged molecules in the ECM such as GAGs, thereby influencing cytokines and growth factors that control cell signaling throughout the regeneration process. On the other side, the NO2B6 sample exhibited competitive properties of mechanical strength, porosity, swelling rate, and biodegradability when compared to other groups. It also revealed the more appropriate macropore as well as micropore size for the in vitro evaluation toward the superior cell–biomaterial interaction including cell survival, proliferation capacity, adhesion, and spreading properties on the surface toward the tissue regeneration for further experiments. Thus, the NO2B6 hydrogel group would be selected for the following experiments as a promising candidate for the bone regeneration layer along with the NO2 group for the cartilage regeneration toward the bilayer scaffold fabrication for tissue regeneration with the aim of enhancing osteochondral tissue regeneration efficiency.

**Fig. 7.**
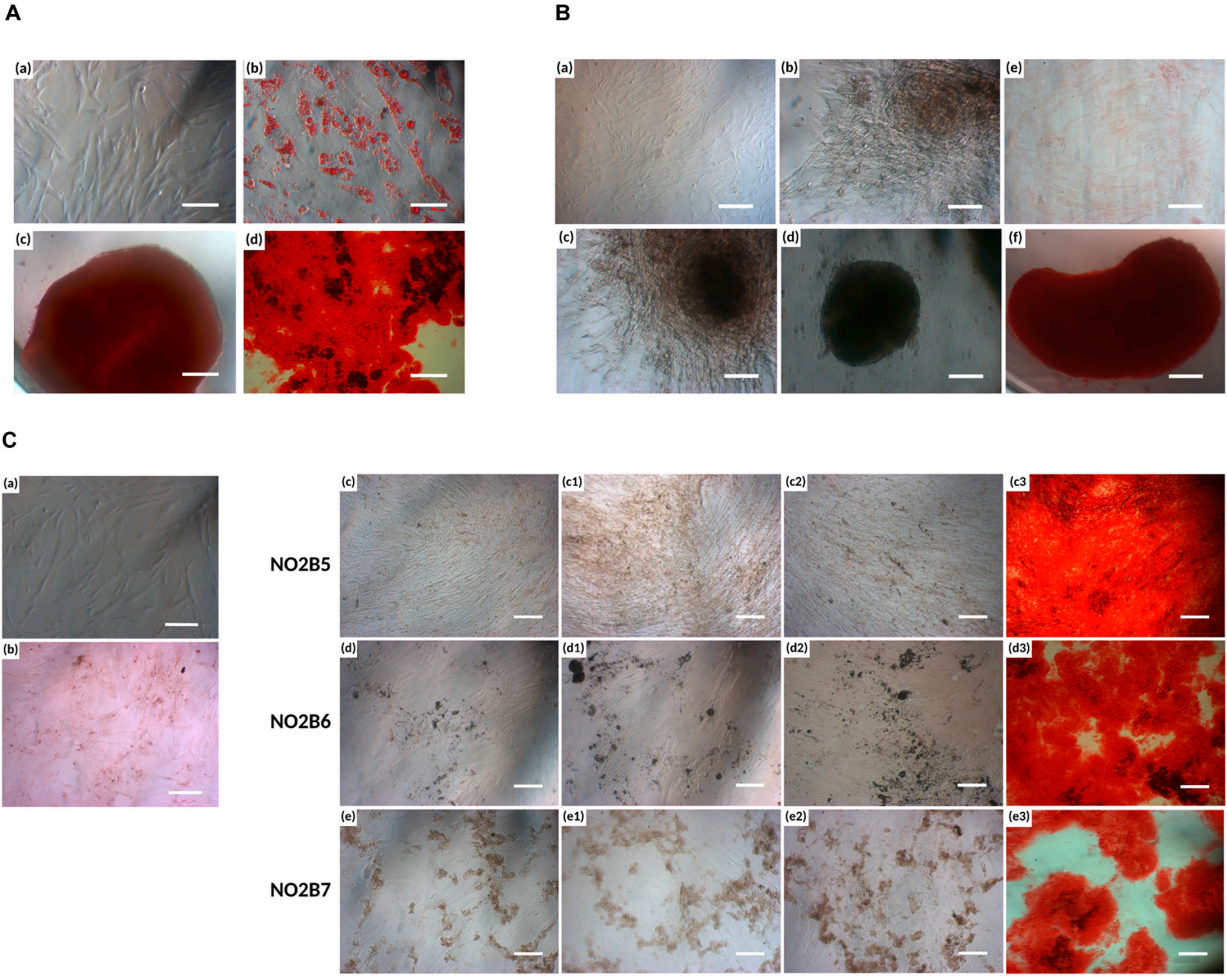
In vitro evaluation. (A) Multilineage differentiation capacity of R-BM cells including (a) R-BM morphology before changing the differentiation medium. Oil Red O (b), Safranin O (c), and Alizarin Red S (d) staining after induction of adipogenic, chondrogenic, and osteogenic differentiation, respectively. (B) Chondrogenic differentiation of R-BM cells cultured in the scaffold extraction and chondrogenic differentiation medium. (a) R-BM cells before changing the differentiation medium; (b, c, and d) R-BMs after 7, 14, and 21 d of changing the cartilage differentiation medium, respectively; (e and f) Safranin O staining of the control group (cells without the induction medium) and cartilage induction differentiation group. Scale bars, 100 μm (a to d) and 200 μm (e and f). (C) Osteogenic differentiation of R-BM cells cultured in a mixture of scaffold extraction and the osteogenic differentiation medium. (a) R-BM cells before changing the differentiation medium; (b) Alizarin Red S staining of control group (cells without the induction medium); R-BM cells cultured with (c, c1, c2, and c3) NO2B5, (d, d1, d2, and d3) NO2B6, and (e, e1, e2, and e3) NO2B7 after 7, 14, and 21 d of changing the cartilage differentiation medium, respectively, and staining with Alizarin Red S. Scale bars, 200 μm (a and b) and 100 μm (c to e).

The morphology and differentiation capacity of R-BM cells are investigated to evaluate their characteristics of bone marrow mesenchymal stromal cell (BMSC). According to the International Society for Cellular Therapy (ISCT), MSCs must be able to differentiate into osteoblasts, adipocytes, and chondroblasts under standard in vitro differentiating conditions. In the beginning, R-BM cells presented spindle-shaped, fibroblast-like cells, which are representative of the characteristics of MSCs. After reaching the confluency of approximately 80%, the medium was replaced with the adipogenic, osteogenic, and chondrogenic differentiation medium corresponding to each type of differentiation. After 21 d of induction, the cell was then stained with a specified solution to detect the signs of differentiated cells. First, Oil Red O was used to reveal their adipogenic differentiation capacity. Through the lipid droplets in the cytoplasm, it suggested the adipogenic differentiation capacity of BMSC under the differentiation condition. For osteogenic differentiation, abundant calcium deposition accumulated on the surface of the well was stained with Alizarin Red S, indicating the osteogenic differentiation ability of MSC. Then, Safranin O confirmed the presence of GAGs, proteoglycans, and collagens after the induction culture after 3 weeks, posing the chondrogenic differentiation of MSC (Fig. 7A). These results suggest that R-BM cells possessed the characteristics of BMSCs [34].

After confirming the characteristics of BMSC, R-BM cells were used to culture with the hydrogel extraction under the chondrogenic and osteogenic induction medium to investigate the chondrogenic and osteogenic differentiation capacity of the scaffold compared to the control group. Scaffold extraction was used by incubating the lyophilized samples for 24 h at 37 °C in the cell culture medium as have been mentioned before. Simultaneously, hydrogel extraction and the differentiated induction medium were combined within the 1:1 volume ratio and replaced every 3 d to induce the chondrogenic differentiation for the NOCC-OXG hydrogel group and osteogenic differentiation for the NOCC-OXG-BCP group along with 3 ratios of the BCP content mentioned above. Cell morphology was observed at days 7, 14, and 21 and then stained with Safranin O for GAG production and Alizarin Red S for calcium deposition after determined intervals. After 7 d of chondrogenic-induced differentiation, cell proliferation appeared to decrease and started to enter the chondrogenic differentiation process, as shown in Fig. 7B. Cells lose their shape of fibroblast-like elongated shape, starting to adopt toward the round shape of chondrocytes. The cell aggregation process into the small and dense cell cluster occurred as the result of the beginning of the chondrogenic differentiation process, followed by the synthesis and deposition of ECM components on the surface of the well, which played a critical role in the next days. After 14 d of culturing, cells exhibited more pronounced characteristics of chondrocytes, indicating their progression toward cartilage-like phenotype. Cells were remarkably observed under the microscope within more defined spherical shape and dense ECM formation, creating a more defined and structured matrix like native tissue. Higher levels of GAGs were synthesized, contributing to the gel-like matrix properties and further supporting the chondrogenic phenotype. At 21 d of chondrogenic differentiation, cells exhibited mature characteristics of chondrocytes, including a spherical shape of chondro-like structure, and rich ECM production without the detection of a single cell. The Safranin O staining process marked the successful chondrogenic differentiation of cells under the induction medium after 21 d when compared to the control group. By the staining method, strong, uniform red staining indicated successful chondrogenesis with a rich deposition of GAG formation, while little differentiated cells with GAG components were seen in the control group without the differentiation medium. In comparison with the culturing of R-BMs within the chondrogenic-induced medium, the amount of matrix detected on the surface of the well did not diminish but was expressed at a higher level. This result could emphasize the ability of the NOCC-OXG bioink to simultaneously support the chondrogenic differentiation capacity of R-BMs within the differentiation-induced medium toward the corresponding tissue for cartilage regeneration.

Similar to the chondrogenic differentiation of the NOCC-OXG scaffold group, NOCC-OXG-BCP scaffold extraction was brought to culture with the osteogenic-induced differentiation medium to examine the osteogenic differentiation capacity within various ratios of BCP contents. Observation of cell differentiation was conducted at 7, 14, and 21 d and then stained with Alizarin Red S to qualify the calcium deposition observed on the well surface. Unlike the chondrogenic differentiation process, cells in this case still retained the elongated or spindle shape, representative of the characteristics of pre-osteoblast cells. Entering the differentiation process, the cell proliferation rate may decrease; instead, cells become more densely packed and concentrated in flow. On the surface of NO2B6 and NO2B7 samples, the early sign of mineralization existed, where cells began to deposit calcium and phosphate on the surrounding surface, which might later play an important role in formation of HAp crystals. Changes started to be prominent on days 14 and 21 with the densely packed cell concentration and highly confluent area. The multilayer formation of cells appeared in all 3 groups. Substantial mineral deposits were visible, with extensive calcium and phosphate incorporation on the well’s surface. The appearance of calcium and phosphate deposits on the surface was detected by Alizarin Red S staining solution, which investigated the osteogenic differentiation support of various BCP contents under fixed differentiated induced medium culture conditions. As reported in Fig. 7C, a major difference existed between the control group (culturing of R-BMs without the differentiation induction medium) and hydrogel groups through the bright red color of staining solution, signifying the presence of calcium and the extent of mineralization, while no calcium deposit was detected on the surface of the control group. When comparing the osteogenic differentiation result of experimental samples to that of Fig. 7A (d), it appeared that the amount of calcium deposit on the surface of the well became more fulfilled over the surface, where it could be inferred that with the supplemented factor into the existing hydrogel system, it had little effect on the differentiation process but enhanced them toward the interest tissues for regeneration. Factors such as concentration, ion release, and surface roughness can differentially impact osteoblast function, viability, proliferation, differentiation, and ECM formation [35]. Free ions released in the vicinity of bone progenitor cells may persist and potentially stimulate osteogenic differentiation, thereby contributing to the new bone formation.

Osteochondral tissue has a complicated structure including the hierarchical and organizational structure of the tissue. Composed of articular cartilage (hyaline cartilage) and a subchondral bone region, each layer has its own ECM composition, with distinct biological and mechanical properties [36]. The monophasic scaffold has been one of the earliest methods for osteochondral tissue regeneration. By the same structure and material composition of the entire scaffold, the monophasic scaffold is applied for bone repair or cartilage repair alone. Otherwise, it does not meet the requirement of osteochondral tissue repair due to its homogeneity [37]. In the utilization of the NOCC-OXG-BCP single-layer 3D-printed scaffold for osteochondral tissue regeneration, the incorporation of BCP may affect the scaffold’s mechanical and biological properties, potentially leading to discrepancies with the native cartilage layer characteristics. Furthermore, the hydrogel system’s ability to support vascularization for subchondral bone regeneration could contribute to cartilage calcification, ultimately compromising the cartilage tissue’s elasticity and damaging the tissue structure [38]. Reversely, the NOCC-OXG-based hydrogel system lacks the ability to provide the osteoconductive property, which plays a crucial role in the osteogenesis process for the new bone formation of the subchondral bone layer. To address limitations, a bilayered scaffold is essential to create distinct platforms that simultaneously support the regeneration of both tissue types. Able to mimic the natural characterization of osteochondral tissue, it can provide the most optimized characterization for each tissue type’s regeneration process, including mechanical properties and biological properties such as porosity, swelling rate, and degradation rate. Moreover, the integration of different biomaterials of different layers serves for the different layer-specific cell support, which are chondrocytes for chondrogenesis and osteoblasts for osteogenesis, and selective biological cues for better regeneration process [39].

In assessing biomaterial strategies of in vivo model for osteochondral tissue repair, the selection of an appropriate animal model is crucial to ensure relevance to human clinical applications, particularly concerning biological properties and cartilage physiology. A wide range of animal models have been considered for this purpose. In this study, the rabbit model was selected due to its numerous advantages, including cost-effectiveness, ease of handling, and simple housing requirements [40]. The rabbit defect model has been extensively utilized in research, with defects typically created in either the femoral condyle or the trochlear groove [41,42]. Notably, studies have shown that the trochlear groove of the rabbit shares a greater compositional similarity in bone mineral density and bone volume fraction with the human femoral condyle, a common site of cartilage injury in humans [43]. This similarity highlights the rabbit model as a particularly advantageous choice for in vivo experiments, offering valuable insights that are more likely to translate to human clinical settings. The critical size of a defect is a crucial factor that influences the lesion site’s ability to heal [20]. Specifically, it refers to the smallest defect size that an animal cannot heal on its own without intervention, providing an objective measure of the scaffold’s effectiveness in promoting regeneration [40]. Additionally, a defect size of 3.0 × 3.0 mm is commonly employed in previous studies to establish a full-thickness defect that is suitable for evaluating osteochondral regeneration [40,44]. Therefore, in this study, rabbit models were used to design the 3.0 × 3.0 mm size of defect on rabbit’s trochlea groove. Experimental samples including healthy, untreated, bilayered hydrogel grafting (BHG) and OAT were examined through gross visualization, x-ray observation, and histological analysis for different healing efficacy evaluation.

**Fig. 8.**
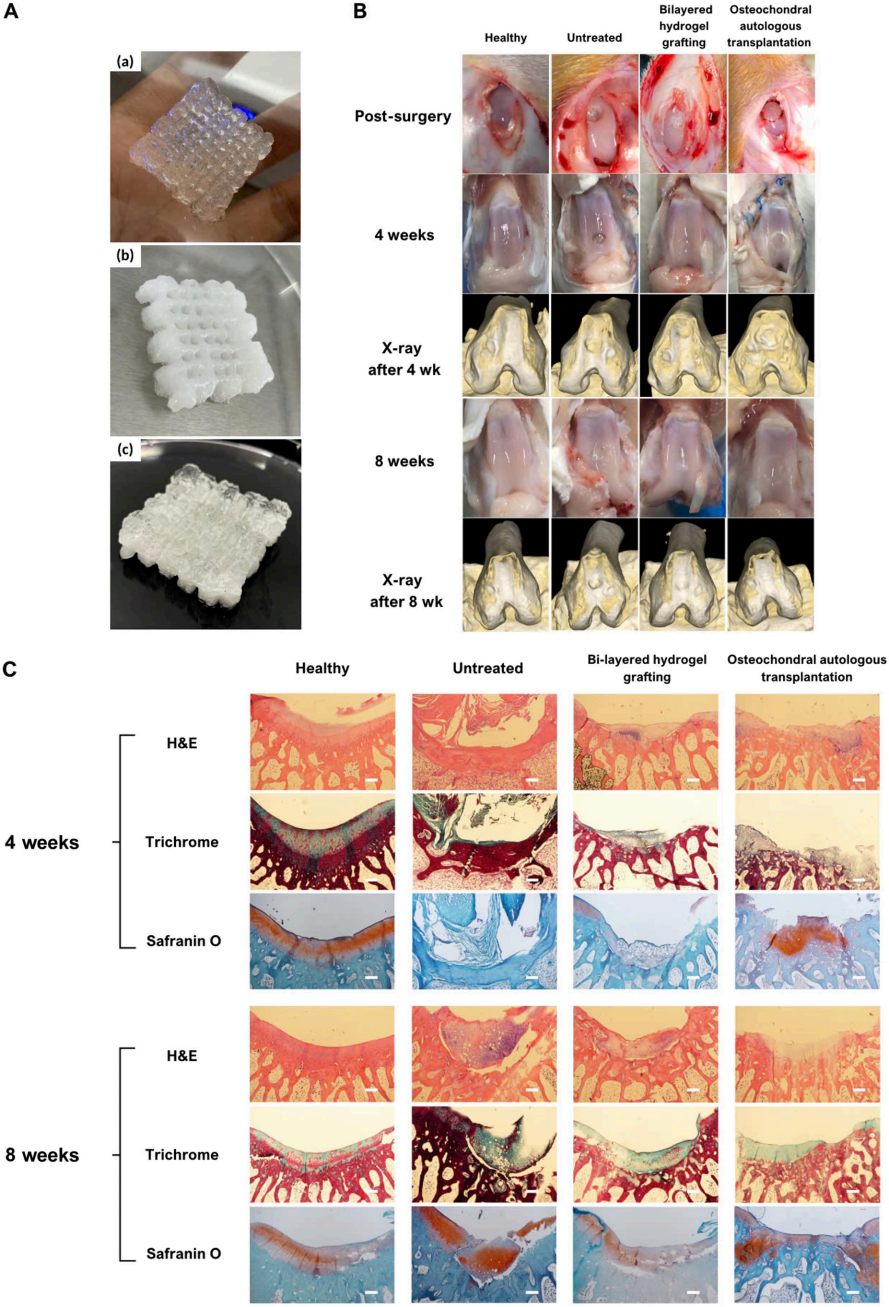
In vivo evaluation. (A) Bilayer scaffold (c) of NOCC-OXG (a) and NOCC-OXG-BCP (b) fabrication. (B) Rabbit defect healing model including healthy, untreated, BHG, and OAT revealed through visual and x-ray images after 4 and 8 weeks. (C) Histological analysis of repaired osteochondral tissue including H&E, trichrome, and Safranin O staining after 4 and 8 weeks. Scale bar, 200 μm.

Osteochondral tissue from healthy rabbits took the responsibility as the positive control group, with the untreated group as negative control, and regeneration capacity was observed between the BHG and OAT group, as shown in Fig. 8. Apparently, through the surgery defect, there was no inflammatory response, and no scaffold delaminated or migrated from the defect into the joint cavity. After 4 weeks, the untreated group exhibited a delayed regeneration process, evidenced by a distinct indentation on the wound surface. In contrast, the OAT and BHG scaffold groups demonstrated accelerated healing rates comparable to those observed in healthy samples. Visually, neotissues have developed over the wound defect, seamlessly integrating with the surrounding tissue. Nevertheless, the presence of a white circular shape around the wound surface at week 4 indicated ongoing regeneration, corroborated by an evident concave shape and boundaries between neotissue and adjacent native cartilage visible through the gross appearance and x-ray images. According to histological analysis, clearer analysis of each layer regeneration was evaluated, providing a more accurate perspective on the tissue repair efficiency of each scaffold. Through H&E staining, native articular cartilage is surrounded at the top by a weak-stained pink layer of connective tissue known as the perichondrium, followed by the subchondral bone with darker pink color and bone tissue with hollow at the bottom, demonstrating the expression of healthy tissue [45]. At the 4-week mark post-implantation, BHG demonstrated clearer regeneration of the cartilage tissue layer at the surface compared to OAT implantation, exhibiting more distinct layers in deeper regions. However, the regenerative tissues exhibited irregular shape and uneven distribution of tissue’s depth compared to healthy tissue, suggesting an incomplete healing process. Image analysis using Masson’s trichrome and Safranin O staining provided a clearer depiction of the little appearance of the cartilage tissue formation after 4 weeks. Staining with Masson’s trichrome, a smooth and intact surface with hyaline articular cartilage was observed by the dense green color observed on the surface of healthy tissue [46]. With the nonsupportive treatment, the untreated group revealed the new tissue forming with no arrangement, with little detected staining solution observed on both trichrome and Safranin O images. Besides, neither experimental sample showed significant collagen matrix formation on the defect site's surface, indicating limited articular cartilage tissue development. Simultaneously, the appearance of the dense orange-red layer on the top of tissue, which is specifically representative for the GAG content of cartilage tissue, was reflected evidently at the top of the tissue on the healthy rabbit model, indicating the presence of an ECM-rich sulfated proteoglycan [47]. The BHG group exhibited the surface of wound site covered with little staining color, marking the pre-formation of the GAG matrix, while the dense orange-red color was obviously exhibited at the defect site of OAT, illustrating the superior process of cartilaginous formation due to its superiority of tissue’s origin, but not evenly distributed over the surface. This result could be explained by the stages of the new cartilaginous tissue formation. Collagen serves as the initial framework to provide structural support and stability upon which the new tissue can organize and grow. From that, the production of GAG matrix follows [48]. Therefore, in this situation, the early signal of Masson’s trichrome staining was demonstrated over the surface of the wound defect marking the early event in cartilage tissue regeneration, paving the way for subsequent GAG matrix deposition.

At week 8, all the wounds healed almost entirely, where neotissues began to cover into the repair area, while the incomplete process of integration of the neotissues into the adjacent native tissue appeared along with scaffold graft degradation when compared to the healthy sample [49]. Healthy rabbits illustrated the expected healing target after the 8-week healing process, along with the clear visualization of the organization within the cartilage and bone, followed by the collagen components and GAG matrix formation. Generally, BHG and OAT were entering the last stage of cartilage tissue formation, demonstrating the completion of the healing process. In both groups, the thick layer of cartilage was denoted by the strong staining solution attached to the surface of the defect site, although not evenly distributed cartilaginous formation was seen on the treatment site of the OAT sample. Subchondral bone has replaced some of the tissues in the deeper portion of the BHG sample’s repair tissue, imitating the histological architecture of the surrounding tissues. This suggests that a strong healing process is taking place between the repair and the surrounding tissues. Healing site revealed a larger layer of perichondrium, mainly distributed in the middle of the tissue, with the clearer layer of subchondral bone when compared to that of week 4; however, it was still not comparable to healthy tissue. The same result was observed at the OAT site, although a thinner perichondrium layer existed. Simultaneously, the results of Masson’s trichrome and Safranin O staining assays indicated the formation of various collagen and GAG components across the wound surface. Regeneration at week 8 proceeded better with the dense blueish and orange staining color, evenly distributed throughout the surface of the treatment site of the BHG sample, while lower efficacy of the healing capacity of the OAT sample was shown through the heterogenous distribution, demonstrating the superior effectiveness in supporting cartilage regeneration of the hydrogel-based scaffold.

Overall, the BHG demonstrated superior efficacy in bone–cartilage regeneration compared to the OAT group, although the healing process remained ongoing beyond the eighth week. Gross visualization, x-ray imaging, and histological analysis revealed that the scaffold facilitated the formation of comparable collagen and GAG content, as well as the development of distinct layers of cartilage and subchondral bone tissue, closely resembling that of normal tissue. In this study, 3D printing leverages the advantage of BHG by selectively combining biomaterials tailored for osteochondral tissue regeneration, considering the inherent complexity of the tissue. The bilayered scaffold fosters the integration between 2 distinct layers, maintaining separate yet seamlessly connected regenerative processes. By the foundation of the natural hydrogel of NOCC-OXG, it creates an optimal cartilage-like microenvironment, promoting chondrocyte growth and differentiation and thereby facilitating chondrogenesis and the formation of new cartilage tissue. Moreover, incorporating the BCP component into the hydrogel system not only strengthens the scaffold’s mechanical properties but also provides osteoconductive features that support osteoblast growth and differentiation, thereby promoting osteogenesis through the stimulation of bone-specific protein secretion, followed by the seamless facilitation of cartilage tissue repair through the underlying vascular network.

While this study demonstrates promising outcomes, certain limitations must be acknowledged. Although mechanical and biological experiments were conducted on the single-layer scaffold, further investigation of bilayered scaffolds is essential for a more comprehensive evaluation of their transplantation efficacy. In vivo degradation studies should be conducted to accurately determine the degradation rate, considering the scaffold’s interactions with native tissues and the surrounding microenvironment post-implantation. Furthermore, long-term in vivo studies with larger sample sizes are necessary to assess the prolonged effectiveness of 3D-printed scaffolds and establish statistical significance under varying conditions in osteochondral tissue regeneration.

## Conclusion

To conclude, this study has successfully fabricated the bilayered hydrogel-based scaffold with 2 layers for bone–cartilage tissue regeneration along with the combination of NOCC, OXG for cartilage, and BCP supplementation for new bone growth. The successful regeneration by the bilayered scaffold illustrated through the in vivo rabbit wound defect was conducted through the optimization and modification of each type of biomaterial as well as the parameters during the printing process. Through the experiments evaluating the physical and biological properties of experimental scaffolds based on the characteristics of corresponding tissues, selection of optimal oxidation level of OXG and volume ratio between the NOCC-OXG scaffold has been given out to serve as the layer of cartilage tissue regeneration, in particular, $n_{\text{oxidation agent}}$:$n_{\text{xanthan}\;\mathrm{gum}}$ is 2:1 and $V_{\mathrm{OXG}}$:$V_{\text{NOCC}}$ = 2:1. Furthermore, BCP powder concentrations of 50%, 60%, and 70% were selected to serve for the subsequent 3D-printed scaffold process. Cube-shaped design with parameters of 1 × 1 × 1 cm^3^, 0.3 mm layer height, 35% infill percentage, and rectilinear internal infill pattern was successfully fabricated by the pneumatic extrusion-based pressure within the pressure from 1.8 to 3 MPa and printing speed of 650 mm/min, G22 syringe head to achieve the balance between physicochemical properties, cell activities, and vascularization of the scaffold. No cytotoxic effects were observed, while the NO2B6 sample exhibited superior support for cell–biomaterial interactions, enhancing cell proliferation, attachment, and spreading on the 3D-printed scaffold surface. In an in vivo rabbit defect model, the 3D bilayered hydrogel-based scaffold NO2-NO2B6 achieved the best outcomes, proved the effectiveness in mimicking the native osteochondral tissue, and promoted the considerable new cartilaginous tissue formation and complete subchondral bone regeneration, indicating its potential as a promising technique for osteochondral tissue regeneration.

## Data Availability

The data that support the findings of this study are available from the corresponding author upon reasonable request.
