# Supplementary material for: Fabrication of 3-Dimensional-Printed Bilayered Scaffold Carboxymethyl Chitosan/Oxidized Xanthan Gum, Biphasic Calcium Phosphate for Osteochondral Regeneration
Source: Biomater Res. 2025 Apr 9;29:0186. doi: 10.34133/bmr.0186 (PMC11979342; doi:10.34133/bmr.0186)
Supplement: Supplementary 1 — Table S1 [file bmr.0186.f1.doc]

**Front Matter**

Title

Fabrication of 3D printed bi-layered scaffold based on *N,O*-carboxymethyl chitosan/oxidized xanthan gum, biphasic calcium phosphate for osteochondral tissue regeneration

**Authors**

My N-H. Nguyen^1,2^†, Binh T. Vu^1,2^†, Dung M. Truong^3^, Thanh D. Le^4^, Thanh-Tuyen T. Vo^1,2^, Toi V. Vo^1,2^, and Thi-Hiep Nguyen^1,2*^

**Affiliations**

^1^Tissue Engineering and Regenerative Medicine Department, School of Biomedical Engineering, International University, Ho Chi Minh City, Vietnam.

^2^Vietnam National University, Ho Chi Minh City, Vietnam.

^3^Biotechnology Center of Ho Chi Minh City, Vietnam.

^4^Thong Nhat Hospital, Ho Chi Minh City, Vietnam.

† These authors contributed equally to this work

^*^Address correspondence to: nthiep@hcmiu.edu.vn

**Table S1.** Elements detected by EDS technique of NO2 and NO2B scaffold samples

| Element *(Unit: %)* | NO2 | NO2B5 | NO2B6 | NO2B7 |
| --- | --- | --- | --- | --- |
| C | 67.12 ± 0.08 | 27.44 ± 0.14 | 28.49 ± 0.05 | 22.19 ± 0.05 |
| O | 31.09 ± 0.27 | 39.68 ± 0.14 | 36.14 ± 0.19 | 37.04 ± 0.16 |
| Ca | Nd | 20.07 ± 0.13 | 21.97 ± 0.17 | 25.55 ± 0.16 |
| P | Nd | 12.22 ± 0.24 | 13.20 ± 0.35 | 15.35 ± 0.30 |
